# Wnt pathway inhibition with the porcupine inhibitor LGK974 decreases trabecular bone but not fibrosis in a murine model with fibrotic bone

**DOI:** 10.1093/jbmrpl/ziae011

**Published:** 2024-01-21

**Authors:** Hsuan Lung, Kelly L Wentworth, Tania Moody, Ariane Zamarioli, Apsara Ram, Gauri Ganesh, Misun Kang, Sunita Ho, Edward C Hsiao

**Affiliations:** Department of Medicine, Division of Endocrinology and Metabolism, The Institute for Human Genetics, and the Eli and Edythe Broad Institute for Regeneration Medicine, University of California, San Francisco, CA 94143, United States; Oral and Craniofacial Sciences Graduate Program, School of Dentistry, University of California, San Francisco, CA 94143, United States; Department of Dentistry, Kaohsiung Chang Gung Memorial Hospital and Chang Gung University College of Medicine, Kaohsiung 833, Taiwan; School of Dentistry, Institute of Oral Medicine, College of Medicine, National Cheng Kung University, Tainan 701, Taiwan; Department of Medicine, Division of Endocrinology and Metabolism, The Institute for Human Genetics, and the Eli and Edythe Broad Institute for Regeneration Medicine, University of California, San Francisco, CA 94143, United States; Department of Medicine, Division of Endocrinology and Metabolism, University of California, Zuckerberg San Francisco General Hospital, San Francisco, CA 94143, United States; Department of Medicine, Division of Endocrinology and Metabolism, The Institute for Human Genetics, and the Eli and Edythe Broad Institute for Regeneration Medicine, University of California, San Francisco, CA 94143, United States; Department of Medicine, Division of Endocrinology and Metabolism, The Institute for Human Genetics, and the Eli and Edythe Broad Institute for Regeneration Medicine, University of California, San Francisco, CA 94143, United States; Department of Orthopaedics and Anesthesiology, Ribeirao Preto Medical School, University of Sao Paulo, Sao Paulo (SP) 14049-900, Brazil; Department of Medicine, Division of Endocrinology and Metabolism, The Institute for Human Genetics, and the Eli and Edythe Broad Institute for Regeneration Medicine, University of California, San Francisco, CA 94143, United States; Department of Medicine, Division of Endocrinology and Metabolism, The Institute for Human Genetics, and the Eli and Edythe Broad Institute for Regeneration Medicine, University of California, San Francisco, CA 94143, United States; Oral and Craniofacial Sciences Graduate Program, School of Dentistry, University of California, San Francisco, CA 94143, United States; Oral and Craniofacial Sciences Graduate Program, School of Dentistry, University of California, San Francisco, CA 94143, United States; Department of Medicine, Division of Endocrinology and Metabolism, The Institute for Human Genetics, and the Eli and Edythe Broad Institute for Regeneration Medicine, University of California, San Francisco, CA 94143, United States; Oral and Craniofacial Sciences Graduate Program, School of Dentistry, University of California, San Francisco, CA 94143, United States

**Keywords:** fibrous dysplasia (FD), fibrotic bone disease, Gnas, Gsα, GPCR, G-protein coupled receptor signaling, Wnt pathway inhibition, trabecular bone, osteoblasts

## Abstract

G protein-coupled receptors (GPCRs) mediate a wide spectrum of physiological functions, including the development, remodeling, and repair of the skeleton. Fibrous dysplasia (FD) of the bone is characterized by fibrotic, expansile bone lesions caused by activating mutations in *GNAS.* There are no effective therapies for FD. We previously showed that ColI(2.3)^+^/Rs1^+^ mice, in which G_s_-GPCR signaling was hyper-activated in osteoblastic cell lineages using an engineered receptor strategy, developed a fibrotic bone phenotype with trabecularization that could be reversed by normalizing G_s_-GPCR signaling, suggesting that targeting the G_s_-GPCR or components of the downstream signaling pathway could serve as a promising therapeutic strategy for FD. The Wnt signaling pathway has been implicated in the pathogenesis of FD-like bone, but the specific Wnts and which cells produce them remain largely unknown. Single-cell RNA sequencing on long-bone stromal cells of 9-wk-old male ColI(2.3)^+^/Rs1^+^ mice and littermate controls showed that fibroblastic stromal cells in ColI(2.3)^+^/Rs1^+^ mice were expanded. Multiple Wnt ligands were up- or downregulated in different cellular populations, including in non-osteoblastic cells. Treatment with the porcupine inhibitor LGK974, which blocks Wnt signaling broadly, induced partial resorption of the trabecular bone in the femurs of ColI(2.3)^+^/Rs1^+^ mice, but no significant changes in the craniofacial skeleton. Bone fibrosis remained evident after treatment. Notably, LGK974 caused significant bone loss in control mice. These results provide new insights into the role of Wnt and G_s_-signaling in fibrosis and bone formation in a mouse model of G_s_-GPCR pathway overactivation.

## Introduction

Musculoskeletal disorders contribute significantly to morbidity and include conditions of bone loss and abnormal bone gain^1^. Osteoblastic dysregulation is thought to be a major contributor to these conditions. Although multiple signaling pathways, including G_s_-GPCR and Wnt/β-catenin, have been identified in bone, our limited understanding of how they interact to control bone formation has impeded the development of novel treatments for musculoskeletal disorders.^2^

G protein-coupled receptor (GPCR) signaling mediates a wide spectrum of physiological functions, including bone development and remodeling.^3^^,^^4^ The diversity of GPCRs and their responses to small molecules have made them major targets of over 40% of modern pharmaceuticals.^5^ GPCRs signal through a select number of canonical pathways^6^; the G_s_ and G_i_ pathways increase or decrease intracellular cAMP levels, respectively, by activating or inhibiting adenylate cyclase, while the G_q_ pathway increases intracellular calcium by activating phospholipase C.

The G_s_-GPCR pathway plays a significant role in regulating bone formation.^7^ A prototypical disease of G_s_-GPCR pathway activation is fibrous dysplasia (FD) of the bone.^8^ FD is one of the most common skeletal dysplasias, accounting for 2.5% of all bone lesions and 7% of benign skeletal dysplasias.^9^ FD is a mosaic disease caused by somatic activating mutations in the *GNAS* gene, which encodes the stimulatory G-protein alpha subunit (G_s_α), and leads to constitutive activation of the G_s_ signaling pathway.^10^

Changes in osteoblastic G_s_-GPCR signaling can dramatically affect the bone niche, including hematopoietic stem cell function,^11–13^ fracture repair,^14^^,^^15^ and osteogenic cell fate.^16–20^*GNAS* activating mutations are thought to cause FD bone lesions primarily through increased cAMP production and activation of downstream cAMP signaling pathways.^21^ However, clinical observations suggest that the system is more complex, and that additional signaling factors could exacerbate the growth of lesions. For example, in McCune–Albright Syndrome (MAS),^8^ FD bone can show persistent growth even after the third decade of life, possibly driven by the effects of growth hormone/insulin-like growth factor 1 hyperactivity on FD bone.^22^ Also, patients with Carney’s complex,^23^ caused by activating mutations in the regulatory unit of *PRKAR1A*, which encodes a regulatory subunit of protein kinase A and is downstream of *GNAS*, have hyperpigmented skin lesions and bony abnormalities, yet these lesions differ from those seen in MAS and FD. These observations suggest that *GNAS* activation of other pathways distinct from cAMP/PKA could impact the bone formation process and thus modulate the FD/MAS bone phenotype.

The Wnt signaling pathway is a major regulator of many developmental processes, and Wnt ligands stimulate osteoblast proliferation and maturation.^24^ Wnt signaling plays a critical role in postnatal osteogenic differentiation of mesenchymal stem cells^25^ and is a key regulator in maintaining the balance between osteogenesis and adipogenesis.^26^ Wnt ligands are encoded by 19 *Wnt* genes, which are involved in both canonical and non-canonical signaling. This diversity not only reflects the wide and varied role of Wnts in development and tissue homeostasis but also poses a challenge because of the complexity of signals. The acyltransferase porcupine (PCN) is responsible for palmitoylation of all Wnt ligands in the endoplasmic reticulum.^27^ Since PCN is required for optimal secretion of all Wnt ligands as well as their ability to bind to the frizzled receptor, PCN serves as a potential therapeutic chokepoint in both the canonical and non-canonical Wnt signaling pathways.^28^

Over the past several years, data have emerged showing that *GNAS* can signal and activate ancillary pathways such as Wnt,^18^^,^^19^^,^^20^^,^^29^ Hedgehog,^20^^,^^30^ and Yes-associated protein/Tafazzin (Yap/Taz).^31^ A study on FD bone lesions carrying the G_s_α-activating mutations found increased Wnt/β-catenin signaling in FD bone tissues isolated from patients with FD/MAS.^19^ Additionally, Wnt/β-catenin signaling and bone formation were decreased after the loss of G_s_α. In contrast, expressing the R201H mutant G_s_α protein in osteoprogenitor cells upregulated Wnt/β-catenin signaling and inhibited osteoblast maturation, producing an FD-like phenotype that could be rescued by genetically inhibiting Wnt/β-catenin signaling through *Lrp6* deletion.^18^ Furthermore, the other major Gα proteins (G_i_α/o, G_q/11_α, and G_12/13_α) can also differentially regulate Wnt/β-catenin signaling.^19^ In addition, FD is thought to require cellular mosaicism within a given tissue to develop the disease phenotype.^18^^,^^32^ Despite this knowledge, our understanding of how each individual pathway regulates the clinical or histologic features of FD remains limited.

Here, we use our previously published and well-characterized mouse model that drives G_s_α pathway activity through an engineered G_s_-G-protein coupled receptor (GPCR), Rs1, to test the role of Wnt inhibition on G_s_ GPCR-induced fibrotic bone lesions.^33^^,^^34^ G_s_-GPCR signaling is activated specifically in osteoblastic cell lineages in ColI(2.3)^+^/Rs1^+^ mice using a collagen 1α1 2.3 kb fragment promoter.^34^ Although this model activates G_s_-GPCR signaling at the receptor level rather than at the G_s_-protein level, these mice exhibit a phenotype that shares similarities with the most severe cases of human FD^35^, including dramatic trabecular bone formation, cortical bone loss, fibrotic infiltration of the bone, decreased BMD, and loss of bone marrow space, making this model useful for testing strategies that can mitigate or reverse the phenotype.^36^ We previously showed that discontinuing expression of the Rs1 engineered receptor, thus normalizing G_s_ activation, can lead to dramatic reversal of the FD-like lesions.^37^ Prior whole bone analysis in these mice also showed that several Wnt pathway genes, including *Wnt6*, *Wnt10a,* and *Wnt10b*, appeared to be increased in the FD-like whole bone samples.^38^ Here, we use single-cell RNA sequencing (scRNA-seq) and global Wnt inhibition with the PCN inhibitor, LGK974, in ColI(2.3)^+^/Rs1^+^ mice to delineate how loss of Wnt signaling affects the trabecular bone formation and fibrosis in our FD-like mouse model.

## Materials and methods

### ColI(2.3)^+^/Rs1^+^ mouse model of FD-like bone lesions

All mouse studies were approved by the Institutional Animal Care and Use Committee (IACUC) and the Laboratory Animal Research Center at the University of California, San Francisco. Mice were housed in social groups and fed a normal chow diet for the duration of this study. ColI(2.3)^+^/Rs1^+^ mice were maintained on the FVB/N background and were generated by heterozygote crosses of mice carrying the TetO-Rs1 transgene (MMRRC 030758) with mice carrying the ColI(2.3)-tTA transgene (MMRRC 029992) as previously described.^34^ The Rs1 transgene is an engineered GPCR based on the human serotonin 5HT4b receptor, and expression results in high constitutive G_s_-GPCR signaling activity,^34^ which can be further activated by agonists.^37^ We previously showed that mice maintained on regular chow (LabDiet 5053, PMI Nutrition), to allow for continuous activation of the Rs1 transgene from gestation, developed a dramatic bone phenotype characterized by high trabecular bone formation and fibro-cellular infiltrates, which strongly resemble human FD bone lesions.^34^

### Tissue collection and scRNA-seq

**Figure 1:**
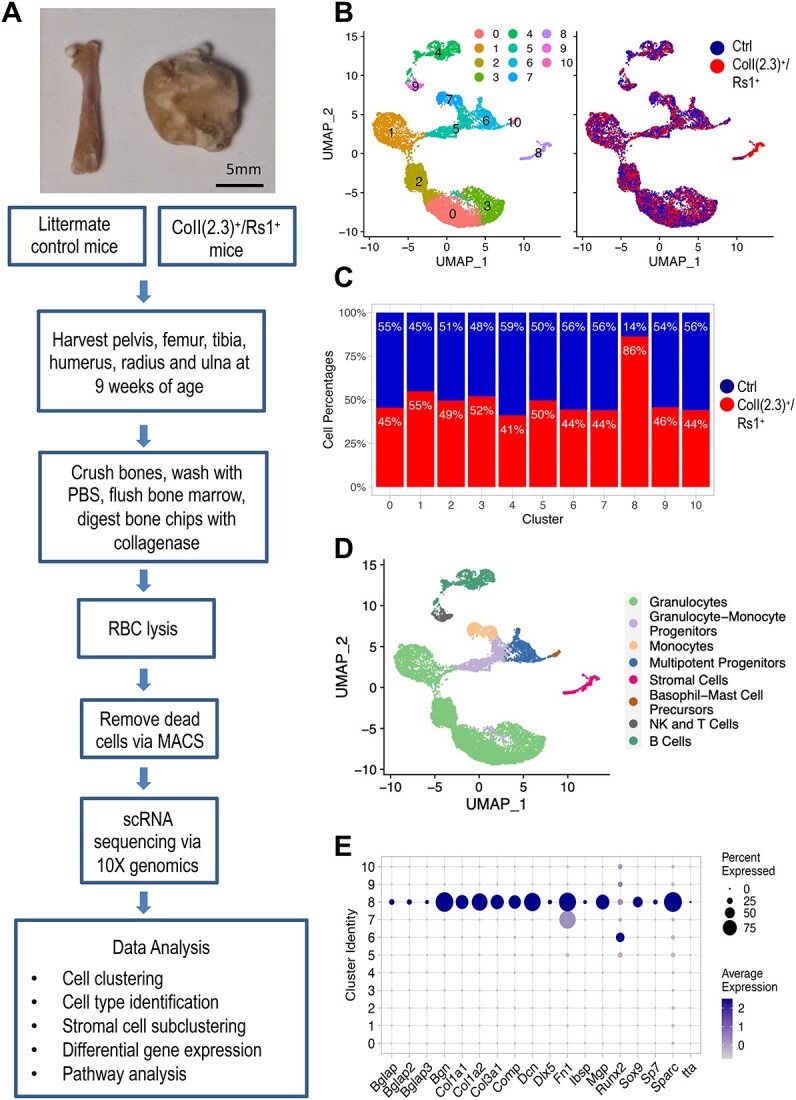
Single cell RNA sequencing (scRNA-seq) of ColI(2.3)^+^/Rs1^+^ mice and littermate controls. (**A**) scRNA-seqtissue processing workflow for ColI(2.3)^+^/Rs1^+^ and control mice. Two 9-wk-old ColI(2.3)^+^/Rs1^+^ and two 9-wk-old control male mice were used for scRNA-seq (see “Materials and Methods”). As shown, the ColI(2.3)^+^/Rs1^+^ mouse femur exhibits significant bony overgrowth, expansion, and obliteration of the typical anatomic landmarks. (**B**) UMAP representation of control and ColI(2.3)^+^/Rs1^+^ cell clusters combined into one Seurat object (left) and split by origin (right). Cells from control bones are depicted in blue and cells from ColI(2.3)^+^/Rs1^+^ bones are depicted in red. (**C**) Percentage of cells from control (blue) and ColI(2.3)^+^/Rs1^+^ (red) bones by cluster. (**D**) Cluster identities for the combined control and ColI(2.3)^+^/Rs1^+^ samples were determined using SingleR. Despite washing the bone marrow fraction, the majority of the cells captured were of hematopoietic origin. Cluster 8 is the stromal cell population. (**E**) Dot plot showing representative expression of known mesenchymal and osteogenic markers in each cluster (0–10) for control and ColI(2.3)^+^/Rs1^+^ samples combined. Expression levels of these genes are highest in cluster 8. Control = ctrl.

Long bones (pelvic girdle, femur, tibia, fibula, humerus, radius, and ulnar bones) were collected from two littermate control (control) and two ColI(2.3)^+^/Rs1^+^ 9-wk-old male mice (Figure 1A) and were gently crushed using a mortar and pestle to release the cells as previously described.^11^ The bone fragments were washed with 1× PBS (Gibco) to separate the hematopoietic cells from the bone chips, which contain the stromal and osteoblastic lineages of interest. The bone chips were incubated in collagenase type I (Worthington Biochemical Corporation, Cat# LS004197, Lot#: 46E16633A), reconstituted in 1× PBS (Gibco) for 60 min at 37°C on an orbital shaker at 110 g. The collagenase was neutralized by adding 5 mL of fetal bovine serum (FBS; Seradigm, Lot# 290B14), centrifuged at 1200 g for 5 min, and resuspended in 2 mL of 2% FBS in PBS. A magnetic-activated cell sorting dead cell removal kit (Miltenyi Biotec,130-090-101) was used per the manufacturer’s instructions to remove the dead cells from the isolated stromal cell population. Single cell libraries were prepared by the UCSF Genomics Core using the 10X Genomics Chromium Single Cell 3′ Kit v.2 according to the manufacturer’s protocol and sequenced on an Illumina NovaSeq 6000. The raw sequencing data were processed using Cell Ranger version 2.2.0 (10X Genomics)^39^ with a modified version of the 10X reference mouse transcriptome (mm 10-1.2.0), which included sequences for the Rs1 and tTA transgenes.^33^^,^^34^ The custom reference was created by incorporating a FASTA file containing the DNA sequences for the Rs1 engineered receptor and the tTA fragment along with an annotation or GTF file for these sequences into the existing mm10 reference using the mkref command in Cell Ranger. The Rs1 and tTA transgenes both include the same rabbit globin intron and polyA sequence in their vectors.^34^ Since 10X Genomics scRNA-seq covers the poly-adenylated 3′ ends of transcripts, this identical sequence in both transgenes creates a multi-mapping issue. Cell Ranger filters out reads that map to more than one locus, so to ensure retention of the rabbit globin intron and polyA reads, we included that sequence only as part of the tTA contig in the custom reference. We use “tTA” as the umbrella identifier for transgene expression. Sequencing files are deposited at the GEO repository (accession number GSE245140).

### scRNA-seq data preprocessing

The gene expression matrices produced by CellRanger for each mouse sample were processed by SoupX (v1.4.5) to remove ambient RNA contamination.^40^ Subsequent quality control steps, as described below, were performed using Seurat (v3.2.2).^41^^,^^42^ Low-quality cells (<200 expressed genes) were removed from sample datasets. Genes expressed in fewer than three cells were also removed. Cells with >5500 or <600 genes were considered outliers with respect to the number of unique genes per cell, suggesting potential doublets or cell fragments, respectively, and were removed. Approximately half of the cells in each of the samples were RBCs; most RBCs were filtered out by removing cells with >100 *Hba-a1* gene transcripts. Low-quality cells identified as having >5% mitochondrial genes in the transcriptome were removed. SingleR (v1.0.1) was used to identify any remaining RBCs^42^ (“erythrocytes”), and these cells were removed. Prior to these quality control steps, the two control and two ColI(2.3)^+^/Rs1^+^ samples contained 7974, 6874, 7463, and 6484 cells, respectively. After filtering, 3441, 2326, 3562, and 2070 control and ColI(2.3)^+^/Rs1^+^ cells were retained for downstream analyses, for a total of 11 399 cells (Table 1). Gene expression data for each cell were normalized using the “LogNormalize” method, where counts for each cell are divided by the total expression, multiplied by a scale factor of 10 000, and then natural-log-transformed using log1p. The 2000 most highly variable genes in each sample were identified using Seurat’s variance-stabilizing transformation or “vst” method for downstream analyses.^41–43^

**Table 1:** scRNA sequencing total cell numbers.

	# of cells	Mean reads/cell	Median genes/cell	# of cells in each cluster (after filtering)												
				Cluster	0	1	2	3	4	5	6	7	8	9	10	Total
CTRL1	7974	67 922	856		798	484	506	450	290	330	294	117	48	99	25	3441
CTRL2	6874	56 569	803		534	264	297	189	366	189	198	196	9	57	27	2326
CTRL Total	14 811			# of cells	1332	748	803	639	656	519	492	313	57	156	52	5767
				Percent (%)	23.1	12.97	13.92	11.08	11.37	9	8.53	5.43	0.99	2.71	0.9	100
ColI(2.3)^+^/Rs1^+^1	7463	49 934	1037		589	643	466	469	244	382	279	118	265	84	23	3562
ColI(2.3)^+^/Rs1^+^2	6484	84 283	798		522	266	321	221	212	129	112	128	94	47	18	2070
ColI(2.3)^+^/Rs1^+^ Total	13 947			# of cells	1111	909	787	690	456	511	391	246	359	131	41	5632
				Percent (%)	19.93	16.14	13.97	12.25	8.1	9.07	6.94	4.37	6.37	2.33	0.73	100
CTRL+ ColI(2.3)^+^/Rs1^+^	28 795			# of cells	2443	1657	1590	1329	1112	11 030	883	559	416	287	93	11 399
Differentially expressed genes per Cluster					**0**	**1**	**2**	**3**	**4**	**5**	**6**	**7**	**8**	**9**	**10**	**Total**
				# of genes	16	6	8	54	40	20	19	47	903	96	337	1546

The baseline cell numbers per sample and per cluster after filtering. The cell numbers and number of differential genes in each cluster (0–10) are also listed. CTRL, control.

### scRNA-seq data analysis

Data integration and cell clustering were performed in Seurat according to the previously published guidelines.^41^^,^^42^ Briefly, after integration of the datasets using canonical correlation analysis (CCA), the expression data for the most highly variable features were scaled so that the mean expression across cells was set to zero. Variables representing the percent of mitochondrial RNA, the percent of ribosomal RNA, and the total number of RNA reads were regressed out against the scaled data. Linear dimensional reduction of the scaled data was performed using principal component analysis (PCA). Clusters were identified using K-nearest neighbor graphing and the Louvain algorithm. The Uniform Manifold Approximation and Projection (UMAP) dimensional reduction technique was used to visualize the clusters. Differentially expressed (DE) genes between different clusters and between experimental groups within clusters were ascertained using a Wilcoxon Rank Sum test. SingleR (v1.0.1), which leverages two reference transcriptomic datasets of pure cell types (ImmGen and Mouse-RNAseq) to infer each cell’s origin, was used to annotate cluster cell types.^44^ To complement SingleR’s cell type annotations, we performed literature searches of the top DE genes in each cluster, checked known cell type marker expression in the clusters, and submitted select expression matrices to the Mouse Cell Atlas for annotation (data not shown).^45^

Subclustering of the stromal cell population was performed by first creating a new Seurat R object containing cells solely from the stromal cluster. Any remaining hematopoietic lineage and endothelial cells were removed. Sample datasets from the combined stromal object were split into their own objects to calculate the most variable genes in each stromal sample separately. Seurat’s SelectIntegrationFeatures method was used to create a new variable features list containing the 3000 top-scoring genes across datasets. The individual sample datasets were merged, and expression data of the previously calculated top 3000 genes were scaled and used as input to PCA. CCA was not used to integrate the samples as described above due to the small number of cells per sample in the subclustered stromal cell object. Integration results using CCA are adversely affected when small datasets are used and when unique cell types between samples may exist. Instead, after merging the stromal samples, the Harmony algorithm was used to remove the influence of dataset-of-origin from the PCA embeddings as demonstrated in Seurat’s Harmony vignette.^46^ Cells were clustered and then visualized as described previously, except that the Harmony-corrected PCA embeddings were used as the dimension reduction parameter. Differential expression analysis was then performed as described above. Single-cell clustering and gene expression data were visualized using the R packages Seurat, scCustomize (v1.1.1), ComplexHeatmap (v2.14.0),^47^^,^^48^ patchwork (v1.1.2), and ggplot2 (v3.4.1).^49–51^ Extracellular matrix (ECM) production was profiled using gene lists compiled in the Matrisome Project (Naba Lab-maintained website: http://matrisomeproject.mit.edu).^52–56^ The matrisome is the ensemble of genes encoding ECM and ECM-associated proteins and is comprised of six gene list categories, namely, glycoproteins, collagens, proteoglycans, regulators, secreted factors, and ECM-affiliated proteins. Biological and functional pathways were identified using ingenuity pathway analysis (IPA) (Qiagen, Inc.).^57^

### Pharmacologic inhibition of Wnt signaling using the PCN inhibitor, LGK974

Four-week-old ColI(2.3)^+^/Rs1^+^ male and female mice were treated with the PCN inhibitor LGK974 (Millipore CAS 1243244-14-5) to inhibit Wnt pathway activity. LGK974 was resuspended in DMSO (5% w/v stock) and dissolved in corn oil to achieve final doses of 5 or 30 mg/kg in 50 μL. LGK974 was administered by oral gavage at either a low dose (5 mg/kg)^58^^,^^59^ for 8 wk or high dose (30 mg/kg)^60^ for 3.5–5 wk for 5 d each wk, starting at 4 wk of age, following published literature. The high dose treatment duration was shorter because of adverse effects, such as weight loss or failure to thrive (data not shown), which triggered early euthanasia for the affected animals following guidance from the UCSF veterinary services. Control animals were either single transgenic or wildtype littermates [WT, TetO-Rs1 single transgenics, or ColI(2.3)-tTA single transgenics] as prior studies indicated no significant differences among these control genotypes or biological sex.^34^ Vehicle-treated mice received 50 μL of corn oil vehicle by oral gavage. All mouse studies were performed following protocols approved by the UCSF IACUC.

### MicroCT skeletal imaging

Bone parameters were assessed at 8 wk of age (high-dose groups) or 12 wk of age (low-dose groups), following established protocols.^34^ Micro X-ray computed tomography (microCT) was performed on formalin-fixed femurs at the indicated time points with morphometric parameters reported in accordance with established standards.^61^ Specimens were imaged at 5 μm/voxel at 55 kVP on a Scanco Medical MicroCT50 scanner at the Imaging Core of the UCSF Core Center for Musculoskeletal Biology and Medicine. One-hundred slices of the mid-diaphysis were analyzed for cortical and trabecular parameters, including total volume (TV, in mm^3^), bone volume (BV, in mm^3^), bone volume-to-tissue volume ratio, or bone volume fraction (BV/TV), trabecular separation (Tb.Sp, in mm), trabecular thickness (Tb.Th, in mm), trabecular number (Tb.N, in 1/mm), structure model index, and connectivity density (Conn.D, in 1/mm^3^). The mean tissue density was calculated by dividing the total bone mineral content by the bone volume (BMC/BV).^34^ The mid-diaphysis was chosen because of the readily available anatomic landmarks that could be used to identify the site despite the malformations caused by the ColI(2.3)^+^/Rs1^+^ transgene. However, ColI(2.3)^+^/Rs1^+^ show little cortical bone, and so assessments were performed following cortical bone parameters for control animals and trabecular assessments for ColI(2.3)^+^/Rs1^+^ animals. High-resolution microXCT was used on the high-dose LGK-treated ColI(2.3)^+^/Rs1^+^ mice because the trabecular structure was below the thresholds of the MicroCT50. We used a MicroXCT-200 (Carl Zeiss Microscopy) at the UCSF Biomaterials and Bioengineering Correlative Microscopy Core, with 10× magnification (spatial resolution: ~2 μm/voxel) and 60 kVp of energy. Tomograms were reconstructed after beam hardening, center shift correction, and CT scaling using XMReconstructor. The same sub-volume (~0.6 mm^3^) of each sample was extracted and segmented to determine BMD (mgHA/cm^3^), bone volume (mm^3^), and trabecular thickness (μm) with AVIZO software (Thermo Fisher Scientific Inc.). BMD was measured based on the calibration curve obtained using known mineral density calibration phantoms.^62^ Bone volume (mm^3^) and trabecular thickness (μm) were determined from the materials statistics and thickness map, respectively. Trabecular number and spacing/connectivity could not be reliably determined from the microXCT data because the trabecular spicules in the treated animals approached the resolution limits of the instrument. Therefore, segment number and branching nodes are reported as measures of trabecular number and connectivity.

Craniofacial bones were imaged on the Scanco MicroCT50 system at 55 kV and 10 μm/voxel with a 0.5 mm aluminum filter. A Gaussian filter was used to suppress image noise: Sigma 5, support 3. For total skull analyses, the lower threshold was 220 mg HA/cm^3^ and the upper threshold was 1000 mg HA/cm^3^. For the trabecular bone analysis of the skull, a lower threshold of 190 and an upper threshold of 1000 was selected. The whole skull was selected as the ROI for whole bone analyses. Variables measured included total volume (TV, in mm^3^), bone volume (BV, in mm^3^), bone volume-to-tissue volume ratio, or bone volume fraction (BV/TV).

For trabecular assessment, a 200-slice ROI was manually contoured, starting 100 slices caudal from the medial aspect of the cranial-most portion of the posterior attachment of the zygomatic arch (Supplemental Figure S1). Parameters calculated included TV, BV, BV/TV, trabecular separation (Tb.Sp, in mm), trabecular thickness (Tb.Th, in mm), trabecular number (Tb.N, in 1/mm), structure model index, and connectivity density (Conn.D, in 1/mm^3^). The mean tissue mineral density (TMD, in mgHA/cm^3^) was calculated by dividing the total bone mineral content by the bone volume (BMC/BV). The thresholds used for all bone analyses were selected qualitatively by an experienced operator (A.Z.) by comparing segmented trabecular bone to original grayscale images, aiming to obtain a physiologically accurate representation. Non-typical thresholds and a Gaussian filter were used due to the specific and rare bone morphology and density known in the ColI(2.3)^+^/Rs1^+^ mice.^36^

### Histology

Femurs were collected, fixed in 10% neutral-buffered formalin for 24 h, and decalcified in 10% ethylenediaminetetraacetic acid (EDTA, pH 7.4). The EDTA was changed five times per week until the bones were pliable (approximately 1 wk for control bones and 3 wk for ColI(2.3)^+^/Rs1^+^ bones). The femurs were processed by the Gladstone Light and Microscopy Core (San Francisco) by embedding in paraffin, sectioning longitudinally at 5 μm thickness, and staining with hematoxylin and eosin (H&E).

### Immunohistochemistry

After deparaffinization of sections mounted on slides, endogenous peroxidase was blocked using 3% H_2_O_2_ for 30 min at room temperature (RT) and nonspecific blocking with 2% goat serum for 20 min at RT. Bones were assessed by immunohistochemistry for numbers of immature osteoblasts, mature osteoblasts, and osteoclasts using primary antibodies against osterix (Sp7) (Abcam, #ab22552), osteocalcin (OC) (Takara, #M173), and cathepsin K (Abcam, #ab19027), respectively. Primary antibodies diluted in PBST/1%BSA and negative control were applied to the sections overnight at 4°C, and secondary antibody (Amersham, NA9340V) was diluted in PBST/1% BSA and applied for 45 min at RT. Positive staining was detected using DAB (3,3′-diaminobenzidine) Substrate Kit for Peroxidase (Vector, Cat# SK-4100) according to the manufacturer’s protocol, and counterstaining was performed with hematoxylin (Mayer, Sigma, 51 275) for 15 s.

### Statistical analyses

Differences between relevant experimental groups were calculated using unpaired, two-tailed *t-*tests (GraphPad Prism). Analyses were considered statistically significant if *P* < .05. Results were described as trending towards significance if *P* < .10, in order to highlight potentially interesting areas for future inquiry.

## RESULTS

### scRNA-seq reveals an expanded stromal cell population in ColI(2.3)^+^/Rs1^+^ long bones

scRNA-seq was performed on cells harvested from the major appendicular bones (pelvic girdle, femur, tibia, humerus, radius, and ulna) of two ColI(2.3)^+^/Rs1^+^ and two control mice (Figure 1A). The bone marrow compartment was flushed to remove hematopoietic cell populations, and a total of 28 795 cells were captured and sequenced across all four samples combined (Table 1). After applying quality control filters to remove doublets, RBCs, and dead cells, a total of 11 399 cells were analyzed (Table 1). The mean reads per cell ranged from 49 934 to 84 283. The median number of genes detected per cell ranged from 798 to 1037. Individual cells from ColI(2.3)^+^/Rs1^+^and control samples were combined into one object, filtered, and clustered using Seurat, as described in the Methods and Supplemental Table S1.

Eleven distinct clusters (0–10) were identified using a shared nearest neighbor–based clustering algorithm with the PCA data reduced to the top 28 principal components (Figure 1B). There was similar representation from ColI(2.3)^+^/Rs1^+^ and control mice in all 11 clusters with the notable exception of cluster 8, which contained a significantly higher percentage of cells from ColI(2.3)^+^/Rs1^+^ (86%) than control (14%) mice (Figure 1C); 6.37% of all ColI(2.3)^+^/Rs1^+^ cells vs only 0.99% of all control cells are located in this cluster (Table 1). SingleR,^44^ an R package that matches gene expression data with existing databases, was used along with known gene markers to identify the representative cell types for each cluster (Figure 1D, Supplemental Figure S2). Despite flushing and washing out the bone marrow fraction, the majority of captured cells were of hematopoietic origin. Granulocytes, monocytes, B cells, NK cells, and stromal cell populations were easily identified, and no significant differences of biological importance were observed in B cells, monocytes, NK cells, granulocytes, or granulocyte–monocyte precursors between ColI(2.3)^+^/Rs1^+^ and control cells (Supplemental Table S2). Cluster 8 was identified as the stromal cell population and was enriched for gene markers of chondrocyte, osteoblast, and fibroblastic cells (Figure 1E), which is consistent with published cell types known to populate murine bone.^63–65^

### Subclustering reveals fibroblastic stromal cell populations comprised primarily of cells from ColI(2.3)^+^/Rs1^+^ bones

**Figure 2:**
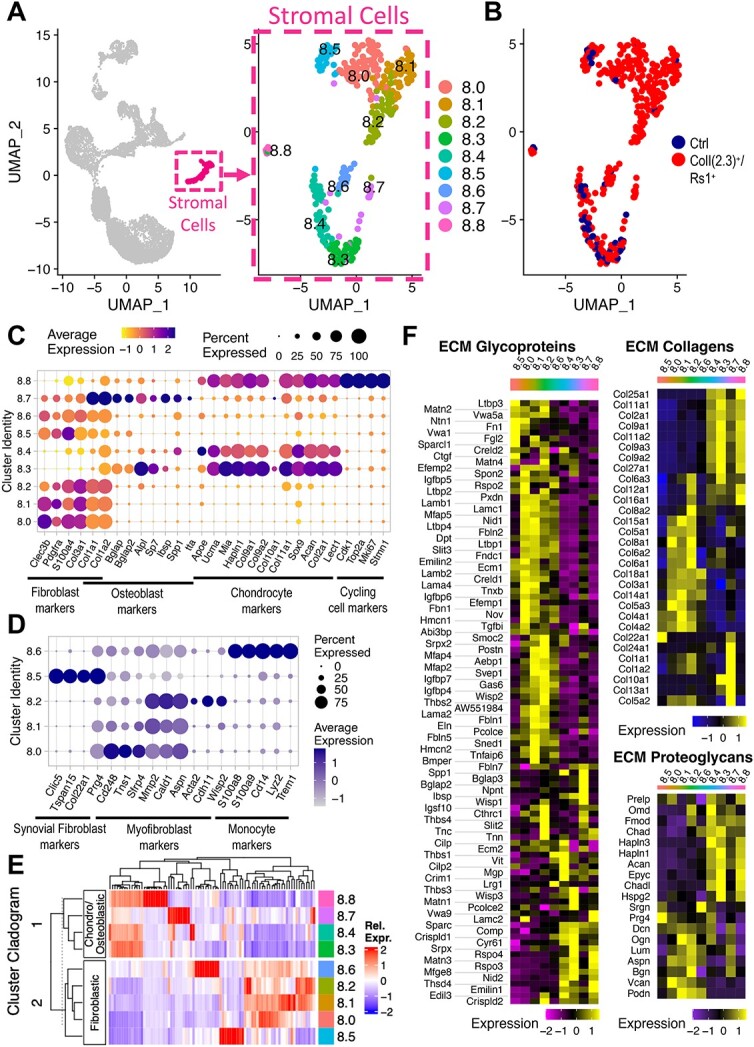
Subclustering of the stromal cell cluster reveals distinct fibroblastic populations. (**A**) UMAP representation of the stromal cell cluster (cluster 8) after subclustering cells into a new Seurat object. Nine distinct subclusters (c8.0–c8.8) were identified. (**B**) UMAP representation of the stromal cell cluster (cluster 8) after subclustering, split by origin (Ctrl = blue; ColI(2.3)^+^/Rs1^+^ = red). (**C**) Dot plot representation of key fibroblast, osteoblast, and chondrocyte markers in the stromal cell subpopulations. Subclusters c8.0, c8.1, c8.2, c8.5, and 8.6 correspond to fibroblastic cells; subclusters c8.3 and c8.4 correspond to chondrocyte subpopulations, including hypertrophic chondrocytes in subcluster c8.3; sub-cluster c8.7 corresponds to osteoblastic lineage cells; and subcluster c8.8 corresponds to proliferating chondrocytes. (**D**) Dot plot representation of fibroblast gene expression levels within the fibroblast subclusters, including synovial fibroblast markers, markers associated with myofibroblasts or that are upregulated during differentiation into myofibroblasts, and monocyte lineage markers. (**E**) Cladogram depicting the relationship between each subcluster of the stromal cell cluster (cluster 8) based on the transcriptomic profiles of each subcluster. The primary branchpoints occur between the chondrocytic/osteoblastic lineages (c8.3, c8.4, c8.7, c8.8) and the fibroblastic lineages (c8.0, c8.1, c8.2, c8.5, c8.6), suggesting that the subclusters within each branchpoint are closely related to each other. (**F**) Heatmap depiction of the core matrisome genes (ECM glycoproteins; ECM collagens, and ECM proteoglycans) expressed in the stromal cell subclusters (c8.0–8.8). The heatmap color blocks depict average gene expression for a given gene across each cluster.

Additional subclustering of cluster 8 was performed to determine if distinct stromal cell types could be further distinguished from each other. Nine subclusters, c8.0–c8.8, were identified after re-clustering (Figure 2A–C). Five of these sub-clusters (c8.0, c8.1, c8.2, c8.5, and c8.6) expressed fibroblastic markers (*Clec3b, Pdgrfa, S100a4, Col3a1, Col1a1*) and were comprised primarily of cells from ColI(2.3)^+^/Rs1^+^ mice with few cells from control mice (221 cells vs 9 cells, respectively) (Figure 2C and Supplemental Table S3). The paucity of cells from control mice in these clusters suggests an expansion of these lineages and is consistent with the abundance of fibroblastic cells observed on histology in ColI(2.3)^+^/Rs1^+^ mice, other murine models of FD, and human FD lesions.^18^^,^^34^^,^^66^^,^^67^^,^^68^ One small sub-cluster (c8.7) was identified based on its high expression of osteoblast markers, including *Col1a1*, *Col1a2, Bglap*, *Spp1*, *Ibsp*, and *Sp7*. This cluster was comprised primarily of cells from ColI(2.3)^+^/Rs1^+^ mice and included cells in which the Rs1^+^ transgene was expressed, as identified by the expression of tTA transcripts (Figure 2C).^34^ The number of cells with detectable tTA transcripts was low in our samples (14 cells total, including 4 cells in subcluster c8.7 [4/19 ColI(2.3)^+^/Rs1^+^ cells = 21%] and 4 cells in the fibroblastic subclusters c8.0, c8.1 and c.8.2 [4/192 ColI(2.3)^+^/Rs1^+^ cells = 2.1%]). Despite the Rs1^+^ transgene being activated in ColI(2.3)^+^ maturing osteoblasts, we saw an expansion of immature, fibroblastic-like cells in the ColI(2.3)^+^/Rs1^+^ mouse bones, suggesting that activation of G_s_-GPCR signaling in maturing osteoblasts could affect earlier developmental lineages in a non-cell autonomous fashion.

Three sub-clusters (c8.3, c8.4, c8.8) expressed high levels of known mouse chondrocyte lineage markers, *Sox9*, *Col2a1*, *Acan,* and *Lect1* (Figure 2C).^69^ Cluster 8.8 expressed both chondrocyte and cell cycle gene markers (*Cdk1, Stmn1, Top2a*, and *Cenpa*) and was determined to represent proliferating chondrocytes. One chondrocyte sub-cluster (c8.4) expressed high levels of *Ucma*, *Cytl1*, *Mgp*, and *ApoE* in addition to *Sox9*, *Col2a1*, *Acan,* and *Lect1*, and is consistent with pre-hypertrophic chondrocytes. A separate chondrocyte sub-cluster (c8.3) expressed high levels *of Col9a1, Col9a2, Mia, Hapln1*, and *Col11a1* in addition to *Sox9*, *Col2a1*, *Acan*, and *Lect1*. A subset of cells from c8.3 also expressed *Col10a1*, which is a marker of hypertrophic chondrocytes. Additionally, the emergence of osteoblastic lineage markers, *Runx2, Alpl, Sp7, Ibsp,* and *Bglap*, is seen in this sub-cluster, highlighting a potential developmental relationship or trans-differentiation between chondrocytes and cells of the osteoblast lineage.^70^

Detailed analysis of the fibroblastic subclusters revealed distinct differences in gene expression within each subcluster, highlighting differences in biological activity (Figure 2D, Supplemental Figure S3A). Pathway analysis of the fibroblast subclusters vs the non-stromal cell lineages using IPA showed upregulation of fibrosis-related pathways (Supplemental Figure S3B). Unsupervised hierarchical clustering was applied to the top differentially expressed genes of the stromal subclusters to visualize the relatedness of the fibroblast subclusters (Figure 2E). The cluster cladogram groups the chondrocytic and osteoblastic lineages together in branch 1, and the five fibroblastic subclusters together in branch 2. Within the fibroblast subclusters, c8.0, c8.1, and c8.2 (c8.0–c8.2) share the highest similarity, followed by c.8.6 and, lastly, c.8.5 (Figure 2E). C8.5 expresses markers associated with synovial fibroblasts (*Clic5, Tspan14, Col22a1, Prg4, Htra1,* and *Has1*), which are present in the synovium of murine joints (Figure 2D).^69^^,^^71^ C8.0–c8.2 express higher levels of genes associated with activated fibroblasts and myofibroblasts, including *Cd248, Tns1, Sfrp4, Mmp2, Cald1, Aspn, Acta2, Cdh11, and Wisp2*, and each of these fibroblast subpopulations secrete different growth factors, morphogens, cytokines, and interleukins (Figure 2F, Supplemental Figure S4A). C8.6 expresses monocyte markers (*S100A8/A9, Cd14, Lyz2, and Trem1*), but similar to c8.0–8.2, also expresses fibroblast and myofibroblast markers (Figure 2D and F; Supplemental Figure S4B).^52^^,^^72^^,^^73^ Myofibroblasts are often derived from fibroblasts that become activated, but can also originate from other cell types including epithelial, endothelial, and smooth muscle cells, as well as monocytes and macrophages.^44^^,^^52^^,^^53^ The shared expression of fibroblast and myofibroblast genes in subclusters c8.0–c8.2 and c8.6 highlights a potential trajectory toward a fibroblast/myofibroblast phenotype from cells of different origins.

ECM production is a hallmark of fibrosis. We profiled the ECM production in our stromal cell subclusters using “core matrisome” and “matrisome-associated” gene sets as previously described.^54–56^^,^^74^ Core matrisome gene sets were grouped into three categories: glycoproteins, collagens, and proteoglycans (Figure 2F). Matrisome-associated gene sets included ECM-affiliated proteins, regulators, and secreted factors (Supplemental Figure S4A). As expected, the chondrocytic and osteoblastic subclusters showed markedly different matrisome expression profiles from that of the fibroblastic subclusters. Interestingly, c.8.0–c.8.2 showed a similar matrisome expression profile to c.8.6 despite c.8.6 having a different expression profile with respect to monocyte marker genes (Supplemental Figure S4B). Differential expression analysis between cells from ColI(2.3)^+^/Rs1^+^ and control mice for the fibroblastic subclusters could not be performed given the low number of cells from control mice.

### Pathway analysis of the stromal cell population predicts activation of the Wnt/β-catenin signaling pathway in fibroblastic cell subclusters

**Figure 3:**
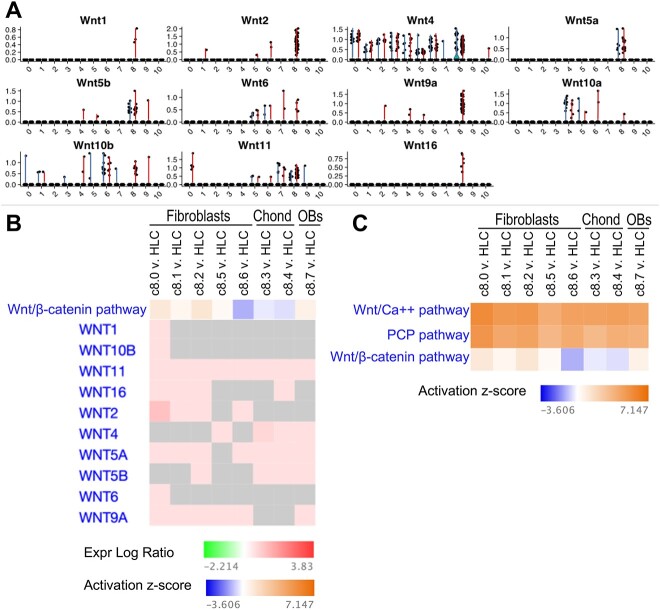
Gene expression of individual Wnt ligands and Wnt/β-catenin pathway activation predictions in control and ColI(2.3)^+^/Rs1^+^ mice. (**A**) Expression levels of individual Wnt ligands in each of the 11 primary clusters, split by control (blue) vs ColI(2.3)^+^/Rs1^+^ (red). Individual Wnt genes were expressed at various levels across the different clusters but were expressed more broadly and at higher levels in cluster 8, the stromal cell cluster, compared to the other clusters, with *Wnt1, Wnt2, Wnt4, Wnt5a, Wnt5b, Wnt6, Wnt9a, Wnt10b,* and *Wnt11* achieving statistical significance (*P*-value <.05). *Wnt2* and *Wnt9a* expression was significantly higher in ColI(2.3)^+^/Rs1^+^ vs control mice in cluster 8 (*P*-value <.05). (**B**) Wnt ligand genes with an FDR <0.05 that are represented in the Wnt/β-catenin pathway in the stromal cell subclusters (c8.0–c8.7, excluding the cycling chondrocytes) compared to the non-stromal hematopoietic lineage cells (HLCs). This analysis was performed using the 194 genes that are associated with Wnt pathway signaling. Genes are represented according to expression log ratio (red = upregulated, green = downregulated, grey = gene is not differentially expressed in that cluster) compared to all other non-stromal HLCs. Wnt/β-catenin pathway activation is represented by the activation Z-score (orange = activated, blue = inhibited, no color = no prediction) when including only differentially expressed genes with an FDR <0.05. (**C** Wnt/Ca^++^, Wnt/PCP, and Wnt/β-catenin pathway activation prediction in the stromal cell subclusters (c8.0–c8.7, excluding the cycling chondrocytes) compared to all other non-stromal, hematopoietic lineage cells, when including the 194 genes involved in Wnt pathway signaling with an FDR <0.05.

To understand which specific cell types in fibrous dysplastic bone might have activated Wnt/β-catenin signaling, we first analyzed the gene expression levels of individual Wnts in ColI(2.3)^+^/Rs1^+^ vs control mice for each of the 11 primary clusters (Figure 3A). Individual Wnt genes were expressed at various levels across the different clusters but were expressed more broadly and at higher levels in cluster 8, the stromal cell cluster, compared to the other clusters, with *Wnt1, Wnt2, Wnt4, Wnt5a, Wnt5b, Wnt6, Wnt9a, Wnt10b,* and *Wnt11* achieving statistical significance (p-value <0.05). *Wnt2* and *Wnt9a* expression was significantly higher in ColI(2.3)^+^/Rs1^+^ vs control mice in cluster 8 (p-value <0.05) (Figure 3A).

We next looked at the expression of individual Wnt genes in the fibroblastic subclusters, which were comprised almost exclusively of cells from ColI(2.3)^+^/Rs1^+^ bones. We performed an analysis using 194 genes that are involved in Wnt pathway signaling and compared the expression of these genes in the fibroblastic subclusters to those in the remaining non-stromal hematopoietic lineage cells (HLCs; Figure 3B; Supplemental Figure S5A–D). The individual Wnt ligands exhibited varying gene expression patterns depending upon the fibroblastic subcluster (Figure 3B). *Wnt9a* and *Wnt11* were upregulated in all of the fibroblastic stromal cell sub-clusters (c8.0, 8.1, 8.2, 8.5, and 8.6) compared to the non-stromal cell lineages, and achieved statistical significance (false discovery rate (FDR) <0.05), while *Wnt2* and *Wnt5a* were significantly upregulated in c8.0, 8.1, 8.2, and 8.6 but not c8.5 (Figure 3B). We then performed pathway analysis using IPA to investigate if the canonical Wnt/β-catenin signaling pathway was dysregulated in the fibroblastic stromal cell populations compared to the non-stromal cell clusters. We observed that the Wnt/β-catenin signaling pathway was predicted to be activated in fibroblast sub-clusters c8.0, 8.1, 8.2, and 8.5 and inhibited in c8.6 (Figure 3C). The Wnt/β-catenin signaling pathway is comprised of a complex network of genes, some of which are anticipated to be upregulated when the pathway is activated and others which are anticipated to be downregulated. Intriguingly, despite a predicted overall positive activation state of the Wnt/β-catenin signaling pathway in sub-clusters c8.0, 8.1, and 8.2, there were a significant number of both up- and downregulated genes in this pathway, including elevated levels of expression of the Wnt inhibitors, *Wif1* and *Sfrp4*. This suggests that compensatory pathways that could inhibit endogenous Wnt signaling might also be activated in these cells (Supplemental Figure S5C and D). The non-canonical Wnt/Ca^++^ and Wnt/planar cell polarity (PCP) pathways were predicted to be activated in the fibroblastic stromal cell subclusters, supporting our hypothesis that both the canonical and non-canonical Wnt signaling pathways may be involved in FD pathogenesis (Figure 3C). These pathways were also predicted to be activated in the osteoblastic stromal subcluster, c8.7 (Figure 3C).

### Low-dose (5 mg/kg/d) LGK974 leads to a mild decrease in trabecular number in established fibrous dysplastic-like trabecular bone in ColI(2.3)^+^/Rs1^+^ mouse femurs

Since the fibroblastic sub-clusters were comprised primarily of ColI(2.3)^+^/Rs1^+^ cells and showed broad Wnt pathway activity, with the exception of c8.6, we hypothesized that ColI(2.3)^+^/Rs1^+^ fibrous dysplastic lesions might be responsive to Wnt inhibition. Since both the canonical and non-canonical Wnt signaling pathways were shown to be potentially activated, we selected to use a PCN inhibitor that would inhibit both canonical and non-canonical Wnt secretion.^58^

LGK974 is a small molecule that inhibits PCN, a Wnt-specific acyltransferase, thereby preventing palmitoylation and secretion of Wnt ligands. It is being tested in clinical trials in Wnt ligand–dependent malignancies (ClinicalTrials.gov #NCT01351103).^75^^,^^76^ Four-week-old ColI(2.3)^+^/Rs1^+^ and littermate control mice were treated with LGK974 at 5 mg/kg/d for 8 wk via oral gavage (Supplemental Figure S6A), and tissue was harvested for histology and microCT imaging. No significant histological changes were identified between vehicle and LGK974-treated control mice (Supplemental Figure S6B). Similarly, ColI(2.3)^+^/Rs1^+^ mice treated with LGK974 did not exhibit significant histological changes compared to vehicle-treated ColI(2.3)^+^/Rs1^+^ mice (Supplemental Figure S6C).

As we have previously shown,^34^ ColI(2.3)^+^/Rs1^+^ mice exhibit significant trabecular bony overgrowth and fibro-cellular infiltration throughout the length of the femur, leading to obliteration of the standard anatomical landmarks used in microCT analysis of the femur (Supplemental Figure S7A and B). To ensure a reproducible site of measurement, we performed microCT imaging at the mid-diaphyseal region, which is easily identifiable in both ColI(2.3)^+^/Rs1^+^ mice and control mice.^77^ Given the paucity of trabecular bone in the distal femur of control mice, trabecular parameters were not computed on control mice, and cortical parameters were used instead. MicroCT evaluation of the mid-diaphysis revealed no significant difference in cortical BV/TV (bone volume/tissue volume) between vehicle and low-dose LGK974-treated control mice (Supplemental Figure S6D and F). There were no significant differences in BV/TV, trabecular thickness (Tb.Th), or trabecular separation (Tb.Sp) between vehicle and LGK974-treated ColI(2.3)^+^/Rs1^+^ mice (Supplemental Figure S6E and G); however, there was a significant increase in trabecular number (*P* = .01) (Supplemental Figure S6G).

### High-dose (30 mg/kg/d) LGK974 leads to significant thinning of established fibrous dysplastic-like trabecular bone in ColI(2.3)^+^/Rs1^+^ mouse femurs

**Figure 4:**
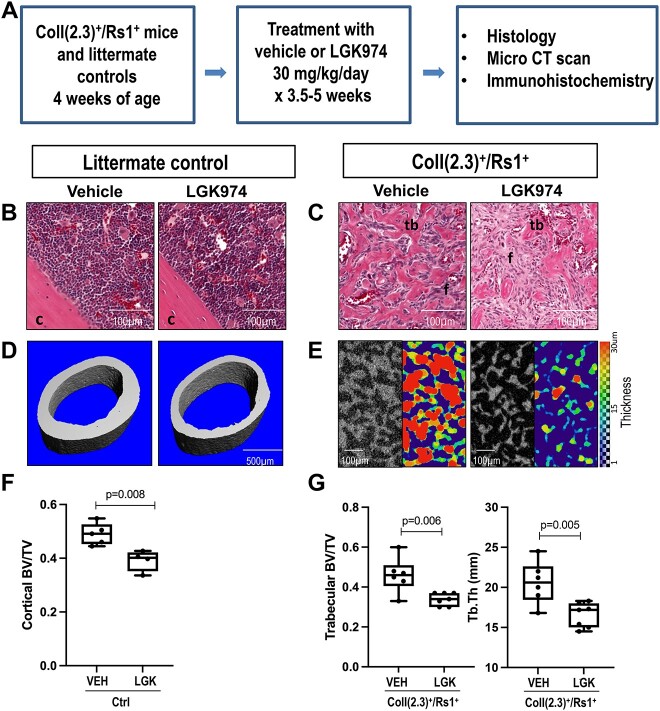
Treatment with high-dose LGK974 (30 mg/kg/d) leads to thinning of fibrous dysplastic-like trabecular bone in ColI(2.3)^+^/Rs1^+^ mice. (**A**) Experimental flow of 4-wk-old ColI(2.3)^+^/Rs1^+^ and control (ctrl) mice, treated with vehicle or LGK974 (30 mg/kg/d) via oral gavage for 3.5–5 wk. (**B**) H&E staining of longitudinal-sectioned control femurs (c = cortical bone). (**C**) H&E staining of longitudinal-sectioned ColI(2.3)^+^/Rs1^+^ femurs shows thinning of the trabecular bone occupying the marrow space (tb = trabecular bone, f = fibrous tissue). (**D**) 3D reconstruction of microCT images of control mid-femoral cross-sections at 5 μm resolution shows thinning of the cortical bone after LGK974 administration. (**E**) High-resolution microXCT images of ColI(2.3)^+^/Rs1^+^ femur at 2 μm resolution show thinning of the trabecular bone. (**F**) Conventional microCT cortical analysis of control mice shows a significant decrease in BV/TV after high-dose LGK974 administration. *N* = 5 control with vehicle, *n* = 4 control with LGK974 (*P* = .008). (**G**) High-resolution microXCT analysis of ColI(2.3)+/Rs1+ mice shows a significant decrease in trabecular BV/TV (*P* = .006) and trabecular thickness (*P* = .005) after LGK974 administration. *N* = 6 ColI(2.3)^+^/Rs1^+^ with vehicle, *n* = 7 ColI(2.3)^+^/Rs1^+^ with LGK974. BV = bone volume; BV/TV = bone volume-to-tissue volume ratio or bone volume fraction; Ctrl = control; VEH, vehicle; LGK = GK974; TV = tissue volume; Tb.Th = trabecular thickness.

To assess if the response to LGK974 was dose dependent, a higher dose of 30 mg/kg/d^60^ was administered to 4-wk-old ColI(2.3)^+^/Rs1^+^ and littermate control mice. The femurs from control mice were harvested for histology and microCT imaging 4.5–5 wk after treatment, while those from ColI(2.3)^+^/Rs1^+^ mice were collected 3.5–5 wk after treatment (Figure 4A). The earlier collection was due to unexplained weight loss in the LGK974-treated ColI(2.3)^+^/Rs1^+^ mice that met pre-specified criteria for euthanasia.

As with the low-dose LGK-treated mice, no major histological changes were observed between vehicle and LGK974-treated control mouse femurs (Figure 4B), and the dense fibrocellular infiltrate characteristic of FD histology remained (Figure 4C). These findings differ from our prior observations where suppression of G_s_-GPCR Rs1 signaling for 16 wk in ColI(2.3)^+^/Rs1^+^ mice^34^^,^^37^ dramatically decreased the abnormal trabecular bone volume, with re-appearance of the bone marrow space and a more defined cortical bone structure.

Three-dimensional reconstruction of microCT scans from control mice treated with LGK974 showed thinning of the cortical bone, as reflected in the significant decrease in BV/TV (*P* = .008) at the mid-femur, which is normally comprised primarily of cortical bone (Figure 4D and F). To better assess the differences in trabecular parameters in the ColI(2.3)^+^/Rs1^+^ mice, which are characterized by an expansion of trabecular bone and significant thinning of cortical bone, we used high-resolution microCT (microXCT), which enabled us to attain a voxel size of 2 μm. ColI(2.3)^+^/Rs1^+^ mice treated with LGK974 showed a significantly lower BV/TV (*P* = .006) and trabecular thickness (*P* = .005) compared to vehicle-treated mice (Figure 4E and G). However, there was no significant difference in bone tissue mineral density (TMD) between these groups (data not shown). The number of mineralized bone segments identified was used as a surrogate for trabecular number (Supplemental Figure S8A), and the number of identified branching nodes was used as a surrogate for trabecular connectivity (Supplemental Figure S8B). These measurements showed a statistically significant decrease in the number of segments identified as well as a decrease in the connectivity, further supporting the assessment that trabecular bone decreased within the LGK974-treated FD-like lesion.

ColI(2.3)^+^/Rs1^+^ mice treated with either vehicle or LGK974 showed persistent expression of osterix (Sp7, immature osteoblasts), osteocalcin (OCN, mature osteoblasts), and cathepsin K (CTSK, osteoclasts) by immunohistochemistry (Supplemental Figure S9A–C). Cells positive for osterix and cathepsin K staining were found lining the bone surface, while OCN was located within the bone matrix. The number of OCN-positive cells was not qualitatively different after LGK974 treatment.

High-dose LGK974 resulted in early lethality in ColI(2.3)^+^/ Rs1^+^ mice; therefore, some mice were collected early at 3.5–5 wk to prevent worsening weight loss and poor mobility. Sub-analyses of mice at 3.5–4 wk and 4.5–5 wk showed a significant decrease in BV/TV (*P* = .02) in the 4.5–5 wk group, but not in the 3.5–4 wk group (Supplemental Figure S10). Although extended treatment with LGK974 might lead to more evident bone resorption in ColI(2.3)^+^/Rs1^+^ mice, potential toxicity of the drug in these mice limited our ability to carry out these longer term experiments.

### High-dose LGK974 does not significantly reduce FD-like bone in the craniofacial skeleton of ColI(2.3)^+^/Rs1^+^ mice

The craniofacial skeleton is significantly affected in FD/MAS patients and in ColI(2.3)^+^/Rs1^+^ mice.^34^^,^^37^ Due to the high regional variability of FD lesions within the craniofacial skeleton, we performed three-dimensional microCT analysis of the whole skulls of the ColI(2.3)^+^/Rs1^+^ mice treated with vehicle or high-dose (30 mg/kg/d) LGK974 to assess for treatment-induced changes (Supplemental Figure S11A and B). No significant differences were seen in cranial BV/TV between vehicle-treated and LGK974-treated ColI(2.3)^+^/Rs1^+^ mice during the treatment period (Supplemental Figure S11C). As noted previously, high-dose LGK974 resulted in early lethality in ColI(2.3)^+^/Rs1^+^ mice. Sub-analyses of these mice at 3.5–4 wk and 4.5–5 wk showed a measurable, albeit not statistically significant (*P* = .108), increase in BV/TV between vehicle-treated ColI(2.3)^+^/Rs1^+^ mice treated for 3.5–4 wk and those treated for 4.5–5 wk (Supplemental Figure S11D). This trend was notably absent in the LGK974-treated ColI(2.3)^+^/Rs1^+^ mice (Supplemental Figure S11D).

**Figure 5:**
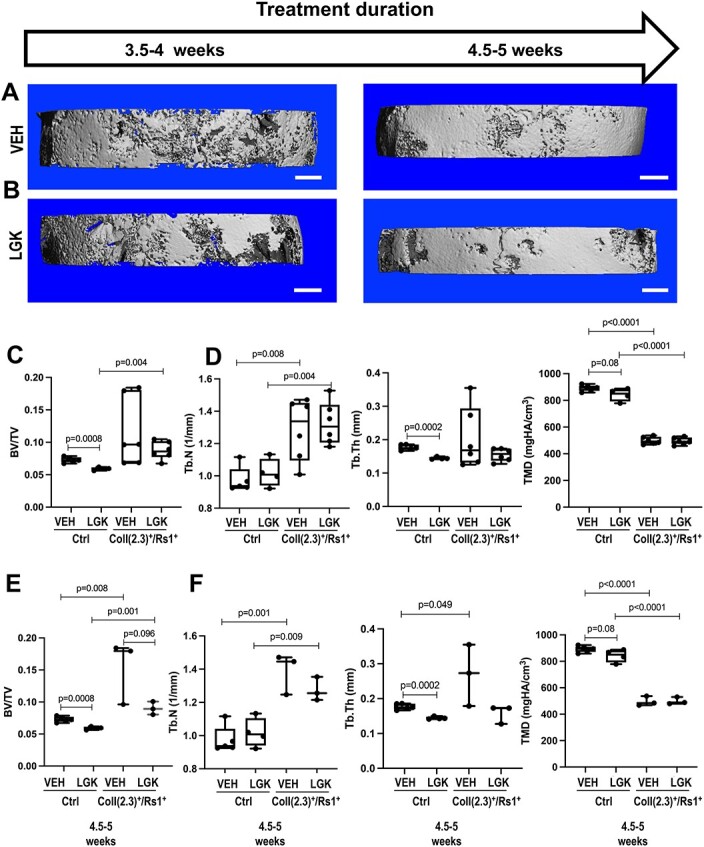
Microstructural assessment of the 200-slice ROI of the skulls from ColI(2.3)^+^/Rs1^+^ mice treated with high-dose LGK974 (30 mg/kg/d). (**A**) Representative images of 200-slice ROI of skulls from vehicle-treated and (**B**) LGK974-treated ColI(2.3)^+^/Rs1^+^ mice at 3.5–4 wk and 4.5–5 wk. The 200-slice segmented ROI images are representative of each time point according to the median microstructural trabecular quantification. The sample size for vehicle-treated ColI(2.3)^+^/Rs1^+^ mice at 3.5–4 wk is *n* = 3, and the sample size for LGK974-treated mice at 3.5-4 wk is *n* = 3. The sample size for vehicle-treated ColI(2.3)^+^/Rs1^+^ mice at 4.5–5 wk is *n* = 3 and the samples size for LGK974-treated ColI(2.3)^+^/Rs1^+^ mice at 4.5–5 wk is *n* = 3. (**C**) LGK974-treated littermate control mice exhibited significantly lower BV/TV (*P* = .0008) than vehicle-treated control mice. There was no difference in BV/TV between LGK974-treated and vehicle-treated ColI(2.3)^+^/Rs1^+^ mice. (**D**) There was no difference in Tb.N between LGK974-treated and vehicle-treated control mice or LGK974-treated and vehicle-treated ColI(2.3)^+^/Rs1^+^ mice. LGK974-treated control mice exhibited significantly lower Tb.Th than vehicle-treated littermate control mice (*P* = .0002). There were no differences in Tb.Th between LGK974-treated or vehicle-treated ColI(2.3)^+^/Rs1^+^ mice. LGK974-treated control mice showed a trend toward lower TMD (*P* = .08) than vehicle-treated control mice. No differences were seen in LGK-treated vs vehicle-treated ColI(2.3)^+^/Rs1^+^ mice. Vehicle-treated ColI(2.3)^+^/Rs1^+^ mice exhibited significantly lower TMD (*P* < .0001) than vehicle-treated control mice, and LGK-treated ColI(2.3)^+^/Rs1^+^ mice exhibited significantly lower TMD (*P* < .0001) than LGK-treated control mice. (**E**) A trend toward a decrease in BV/TV was seen in LGK974-treated vs vehicle-treated ColI(2.3)^+^/Rs1^+^ mice (*P* = .096) when treated for the same duration of time (4.5–5 wk). LGK974-treated littermate control mice had a significantly lower BV/TV than LGK974-treated ColI(2.3)^+^/Rs1^+^ mice (*P* = .001). (**F**) When comparing control and ColI(2.3)^+^/Rs1^+^ mice that were treated for the same duration of time (4.5–5 wk), there were no significant differences in Tb.N between treatment and vehicle in control or ColI(2.3)^+^/Rs1^+^ mice; however, Tb.N was significantly higher between vehicle-treated littermate controls and vehicle-treated ColI(2.3)^+^/Rs1^+^ mice (*P* = .001) and between LGK974-treated littermate controls and LGK974 ColI(2.3)^+^/Rs1^+^ mice (*P* = .009). Tb.Th was significantly higher in vehicle-treated littermate control mice compared to LGK-treated control mice (*P* = .0002) and unchanged between LGK974-treated and vehicle-treated ColI(2.3)^+^/Rs1^+^ mice. Tb.Th was lower in vehicle-treated control mice compared to vehicle-treated ColI(2.3)^+^/Rs1^+^ mice (*P* = .049). TMD remained unchanged between LGK974-treated and vehicle-treated ColI(2.3)^+^/Rs1^+^ mice. BV = bone volume; BV/TV = bone volume-to-tissue volume ratio or bone volume fraction; Ctrl = control; LGK = LGK974; Tb.Th = trabecular thickness; Tb.N = trabecular number; TMD = tissue mineral density; TV = tissue volume; VEH = vehicle.

It was not technically possible to perform detailed trabecular assessment on the whole skull; therefore, we selected a 200-slice ROI caudal to the medial aspect of the posterior attachment of the zygomatic arch to assess trabecular bone microarchitecture in ColI(2.3)^+^/Rs1^+^ and control mice (Supplemental Figure S1, Figure 5A and B). A decrease in BV/TV, Tb.Th, and TMD was seen in LGK974-treated vs vehicle-treated control mice, while no significant changes were seen in LGK974-treated vs vehicle-treated ColI(2.3)^+^/Rs1^+^ mice for any parameters (Figure 5C and D).

Since only a subset of LGK974-treated mice survived to 4.5–5 wk, we next analyzed the mice who were treated for the full 4.5–5 wk duration and compared them to the control mice who were all treated for 4.5–5 wk. Control mice treated with high-dose LGK974 for 4.5–5 wk had a lower BV/TV (*P* = .0008, Figure 5E), Tb.Th (*P* = .0002, Figure 5F), and a trend toward a lower TMD (*P* = .08, Figure 5F) compared to vehicle-treated littermate controls. There were no differences noted in Tb.N (Figure 5F). These findings suggest that LGK974 results in decreased bone volume in the craniofacial skeleton of control mice, similar to what was observed in the femurs, and further indicating the toxicity of LGK974 on the normal skeleton. Conversely, when ColI(2.3)^+^/Rs1^+^ mice were treated with high-dose LGK974, there were no significant changes in any of the trabecular parameters compared to vehicle-treated ColI(2.3)^+^/Rs1^+^ mice (Figure 5F), although a trend toward a reduction in BV/TV was detected (*P* = .096, Figure 5E).

Since treatment response could depend upon treatment duration, we next compared the effects of 3.5–4 vs 4.5–5 wk of LGK974 treatment in ColI(2.3)^+^/Rs1^+^ mice. Similar to our findings in the whole skull, the vehicle-treated ColI(2.3)^+^/Rs1^+^ mice showed a trend toward an increase in BV/TV (*P* = .06, Supplemental Figure S12A) and Tb.Th (*P* = .06, Supplemental Figure S12B) between 3.5–4 and 4.5–5 wk. In contrast, ColI(2.3)^+^/Rs1^+^ mice treated with LGK974 did not display statistically significant changes in BV/TV or other microarchitectural parameters over time (Supplemental Figure S12A–F) with the exception of an increase in TV (*P* = .02, Supplemental Figure S12E). A trend toward a reduction in BV/TV between vehicle and LGK974-treated ColI(2.3)^+^/Rs1^+^ mice over time (*P* = .096, Supplemental Figure S12A) was also noted. Although this could reflect the natural progression of FD-like bone in vehicle-treated mice over time and the potential attenuation of FD-like bone progression in LGK-treated in ColI(2.3)^+^/Rs1^+^ mice, the toxicity of this treatment limited our ability to treat the mice for longer and test this hypothesis.

## Discussion

GPCR signaling is a major regulator of bone formation, yet our understanding of how the downstream pathways impact bone pathophysiology remains unclear. One prototypical G_s_-GPCR disease is FD of the bone, a condition associated with dramatic abnormal and prolific trabecular bone formation accompanied by large numbers of fibroblastic cells within the FD bone lesions.^78^ Our prior studies^34^ and those in other mouse models of G_s_ over-activation^18^^,^^66^^,^^67^^,^^79^ suggest that these fibrotic cells are derived from early osteochondrogenic lineages and may be responsible for the disorganized and expansive trabecular bone seen in FD lesions. In addition, the majority of the FD phenotype was originally thought to be driven directly by the increase in cAMP after activation of the G_s_ pathway,^80^ but data published over the past several years have suggested that ancillary pathways activated by G_s_α, such as Wnt,^18^ Hedgehog,^18^^,^^30^ and Yap/Taz,^31^ are major contributors to the abnormal bone formation in FD. The Wnt signaling pathway is of particular interest due to its established role in developmental processes, including bone and cartilage development.^24^

Three major Wnt signaling pathways have been characterized: the canonical Wnt pathway that signals through β-catenin; the non-canonical PCP pathway; and the non-canonical Wnt/Ca^++^ pathway.^81^^,^^82^ There are 19 known *Wnt* genes in the canonical and non-canonical signaling pathways, and the acyltransferase protein, PCN, is involved in the processing of all Wnt ligands through palmitoylation in the endoplasmic reticulum of Wnt-producing cells.^27^ PCN is required for optimal secretion of Wnt ligands, and can inhibit both the canonical and non-canonical Wnt signaling pathways. Notably, expression of the activating *GNAS^R201H^* mutation in *Prrx1*-Cre mice induces long bone phenotypes reminiscent of FD and impaired mesodermal-derived intramembranous bone formation, both of which could be reversed by blocking Wnt signaling using the PCN inhibitor, LGK974.^20^^,^^82–85^ Despite this suggestive finding, it remained unclear if Wnt blockade would be sufficient to reverse all of the histologic features found in established FD-like bone lesions or only specific parts of the FD phenotype in the setting of overactivated G_s_α activity. In addition, the spectrum of Wnt ligands produced with G_s_-GPCR pathway activation, the cells that produce these ligands, and if direct inhibition of Wnt ligand production could reverse any aspects of an existing FD lesion, remained largely unanswered.

Our study sought to answer these questions using the ColI(2.3)^+^/Rs1^+^ mouse model of FD-like bone in which the G_s_-GPCR pathway is activated in maturing osteoblasts, a narrower set of mature osteoblastic lineage cells than *Prrx1* or other more upstream drivers. Our scRNA-seq analysis revealed several unique findings. First, the cellular compositions of control and ColI(2.3)^+^/Rs1^+^ long bones were surprisingly similar, despite the dramatically different bone phenotypes. Similar to prior reports,^11^ there was a significant increase in the number of stromal lineage cells (cluster 8) in ColI(2.3)^+^/Rs1^+^ mice. This cluster also showed the greatest difference in cell numbers and differentially expressed genes between littermate controls and ColI(2.3)^+^/Rs1^+^ mice. Despite using wash steps to flush out the bone marrow, we still captured a large number of hematopoietic lineages in our scRNA-seq analysis, which reduced our ability to sequence more stromal cells in both the control and ColI(2.3)^+^/Rs1^+^ mice. Subclustering the stromal cell cluster allowed us to identify fibrotic cell populations (clusters c8.0, c8.1, c8.2, c8.5, and c8.6) that appeared to be unique or overrepresented in ColI(2.3)^+^/Rs1^+^ bones, and express markers associated with tissue-resident synovial fibroblasts (c8.5), activated fibroblasts/myofibroblasts (c8.0–8.2, c8.6) and monocytes (c8.6). We speculate that these cell populations are major contributors to the cellular fibrosis characteristic of FD-like bone lesions in the ColI(2.3)^+^/Rs1^+^ mice and may be relevant to the similar cells present in human FD lesions.

Interestingly, the tTA transgene, which was used as a surrogate marker of Rs1 expression, was detected in a smaller percentage of cells than expected in the mature osteoblastic lineage cluster (21%, 4/19 tTA cells). Additionally, tTA transcripts were identified in 2.1% of the cells (4/192 cells) in the fibroblastic clusters, albeit at lower levels than in the osteoblastic cluster. Although scRNA-seq is only able to sample a small number of cells in the total bone, the high level of Rs1 expression observed by qPCR in bone^34^ and prior studies on FACs purified Rs1 expressing cells^11^ suggests that scRNA-seq may underestimate the number of cells expressing Rs1.^34^ This may also indicate that overexpression of Rs1 in osteoblasts^34^ may exert a non-cell autonomous effect on precursor cells, leading to the dramatic phenotype, similar to that observed in other FD models.^18^^,^^32^^,^^66^^,^^67^

Surprisingly, the scRNA-seq results revealed broad expression of many Wnt ligands within multiple cell lineages within the bone, including hematopoietic cells. Previous qPCR expression analysis of whole bone identified that *Wnt6*, *Wnt10a*, and *Wnt10b* gene expression were increased in ColI(2.3)^+^/Rs1^+^ bones but that increased Wnt expression was not clearly detectable in osteoblasts isolated using an FACS gating method.^11^ In our current data, *Wnt1, Wnt2, Wnt4, Wnt5a, Wnt5b, Wnt6, Wnt9a, Wnt 10b,* and *Wnt11* were more highly expressed in the bone stromal cell cluster (cluster 8) than in the other clusters. We observed that the Wnt/β-catenin, Wnt/Ca^++^, and Wnt/PCP pathways were predicted to be activated in many of the fibroblastic sub-types, and this was due to upregulation of multiple Wnt pathway genes, not simply the individual Wnt ligands. This broad expression of Wnt ligands suggested that targeting any one individual ligand may be difficult to interpret due to functional redundancy. LGK974 provided a way to broadly test the role of Wnts within ColI(2.3)^+^/Rs1^+^ bone lesions.

**Figure 6:**
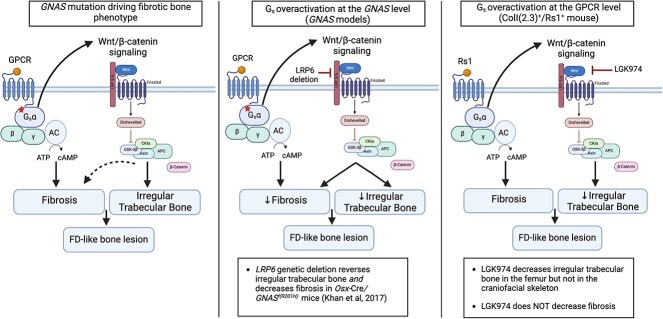
Graphical illustration of the effects of Wnt pathway inhibition in human FD and murine models of FD-like bone. Left panel: graphical depiction of the *GNAS* activating mutation driving the fibrotic bone phenotype in FD. Middle panel: graphical depiction of the effects of Wnt pathway inhibition via *LRP6* gene deletion in the *Osx-Cre/GNAS^f(R201H)^* murine model of fibrous dysplastic-like bone, as previously described in the literature. A decrease in both the irregular trabecular bone and fibrosis is seen. Right panel: graphical depiction of the effects of Wnt pathway inhibition via LGK974 in the ColI(2.3)^+^/Rs1^+^ mouse model with fibrotic bone. A decrease in irregular trabecular bone is seen in the femur but there is no significant reduction in fibrosis. * = location of *GNAS* p.R201C/H mutation in human and murine models of FD; dotted line represents possible pathway connections, solid arrows indicate activation, and solid lines indicate inhibition. Created with BioRender.com.

Treatment with LGK974 decreased trabecular bone volume in the ColI(2.3)^+^/Rs1^+^ mouse femurs, but only at high doses that pushed tolerability. There was a trend toward similar findings in the mouse skulls, and although it did not reach statistical significance, this might have been due to the inability to appreciate changes in trabecular parameters at the standard imaging resolution. Application of higher resolution imaging may be beneficial in future studies. Necropsy on a mouse receiving high-dose (30 mg/kg/d) LGK974 did not reveal any obvious cause of death, and LGK974 was well tolerated in a phase 1 human clinical trial.^59^^,^^75^ It remains possible that the health decline caused by the toxicity of the drug, rather than the direct actions of LGK974 on the skeleton, could have contributed to the overall loss of bone volume, although this is less likely given that bone loss is well documented in clinical trials of LGK974.^75^^,^^76^ Although blocking Wnt signaling significantly decreased the amount of trabecular bone in ColI(2.3)^+^/Rs1^+^ femurs, the bone marrow space remained compromised with large numbers of fibrotic cells. These findings suggest that loss of Wnt signaling was insufficient to reverse fibrosis, but that the irregular trabecular bone formation possibly driven by those cells could be decreased by global Wnt blockade. Since a decrease in abnormal trabecular bone could lead to worsening bone fragility if not coupled by concomitant bone formation, the clinical utility of LGK974 is limited. Our findings also suggest that G_s_-GPCR signaling leads to the expansion of fibrotic cells, that the fibrotic cells produce Wnts that might induce irregular trabecular bone formation, and that blockade of Wnt ligands using LGK974 might only selectively inhibit osteogenesis (Figure 6).

There are several important limitations to our study. First, our scRNA-seq sample size was low due to technical and cost limitations. Although scRNA-seq provides a powerful tool to assess gene expression in mosaic diseases, the relatively low number of cells that could be retrieved, especially from the control bones, and the sequencing depth of 50–80 000 reads/cell, which did not reach full sequencing saturation, limited the detailed analyses in our study. This also limited our ability to compare our findings to other recently published data suggesting the presence of a FD-specific gene expression signature after denosumab treatment.^86^We were only able to identify cells with activated G_s_-GPCR activity from Rs1 based on tTA transgene expression, as discussed in the “Materials and Methods” section. In addition, although the current study identified a role for Wnt pathway activity in driving the FD-like bone phenotype in ColI(2.3)^+^/Rs1^+^ mice, the roles of specific Wnt ligands and how this correlates with human FD bone lesions driven by activating *GNAS* mutations remain unclear.

High doses of LGK974 were required to induce changes in the FD-like bone lesions in our mice, and those changes were not as dramatic as previously seen when Rs1 expression was normalized by suppressing Rs1 expression,^34^ or in other models where G_s_-GPCR pathway activity is normalized.^66^ Additionally, the changes induced by LGK974 were primarily limited to a decrease in BV/TV and trabecular thickness, suggesting that bone resorption was favored over bone formation, which could result in more fragile bone. Dynamic histomorphometry, which would provide a more detailed assessment of bone resorption and bone formation, was not performed on these mice. Furthermore, the fibrotic cell infiltration did not respond to LGK974 treatment. Due to the toxicity of LGK974 on the ColI(2.3)^+^/Rs1^+^ mice, it was not possible to test if a longer treatment duration would lead to ongoing resorption of the irregular trabeculae coupled by healthy trabecular bone formation and a reduction in fibrosis. LGK974 was also associated with significant toxicity to normal bone at this dose, which is concordant with the development of osteoporosis and fractures seen in phase 1 clinical trials of LGK974.^75^^,^^76^^,^^87^ This suggests that inhibiting the Wnt pathway alone is unlikely to be sufficient to fully reverse FD bone lesions, and that directed reversal of G_s_-GPCR pathway hyperactivity, or a combination of pathway inhibitors, may provide more comprehensive reversal of the FD phenotypes. Additionally, although the ColI(2.3)^+^/Rs1^+^ mice exhibit a phenotype that is similar to that of patients with severe FD,^35^ including dramatic trabecular bone formation, cortical bone loss, fibrotic infiltration, and loss of bone marrow space, this model does not fully recapitulate the human disease. Since FD is a mosaic disease and the exact cell types that express the mutation and cause disease in human FD remains unknown, all existing murine models have limitations, and further testing in other murine models of FD should be considered.

Our study yielded several important findings, including the identification of fibroblast cell populations present almost exclusively in ColI(2.3)^+^/Rs1^+^mice, and the complexity of Wnt signaling in the FD-like bone lesions. Broad blockade of canonical and non-canonical Wnt signaling using LGK974, a potent inhibitor of PCN,^58^ partially reversed the abnormal trabecular bone seen in the FD-like lesions in the long bones but did not reduce the fibrotic infiltration. This suggests that Wnt hyperactivity in FD bone^18^^,^^19^ could differentially affect the bone and fibrotic phenotypes present in FD.

## Conclusions

Our scRNA-seq results revealed a surprisingly broad number of cell types that express Wnt ligands in both control and ColI(2.3)^+^/Rs1^+^ mice. In addition, our results showed that broadly inhibiting the Wnt signaling pathway by blocking PCN decreased the trabecular bone volume in established FD-like bone in the ColI(2.3)^+^/Rs1^+^ mouse model. Other features, like the dense fibrotic cell infiltrate, remained unchanged (Figure 6). These findings lay the foundation for future studies to understand how individual Wnt ligands may contribute to FD pathogenesis.

## Supplementary Material

SupplementalFigure1_ziae011

SupplementalFigure2-20240112_ziae011

SupplementalFigure3-20240112_ziae011

SupplementalFigure4-20240112_ziae011

SupplementalFigure5-20240112_ziae011

SupplementalFigure6-20240112_ziae011

SupplementalFigure7-20240112_ziae011

SupplementalFigure8-20240112_ziae011

SupplementalFigure9-20240112_ziae011

SupplementalFigure10-20240112_ziae011

SupplementalFigure11-20240112_ziae011

SupplementalFigure12-20240112_ziae011

JBMR_Plus_SupplementalTables_1_2_3_ziae011

JBMR-Plus-SupplementalFigures_Tables_Legend-20240217_ziae011

JBMR-Plus-SupplementalFigures-20231221_ziae011

## Data Availability

The data that support the findings of this study are available from the corresponding author upon reasonable request.
