# Supplementary figures and images for: Wnt pathway inhibition with the porcupine inhibitor LGK974 decreases trabecular bone but not fibrosis in a murine model with fibrotic bone
Source: JBMR Plus. 2024 Jan 21;8(5):ziae011. doi: 10.1093/jbmrpl/ziae011 (PMC10994528; doi:10.1093/jbmrpl/ziae011)

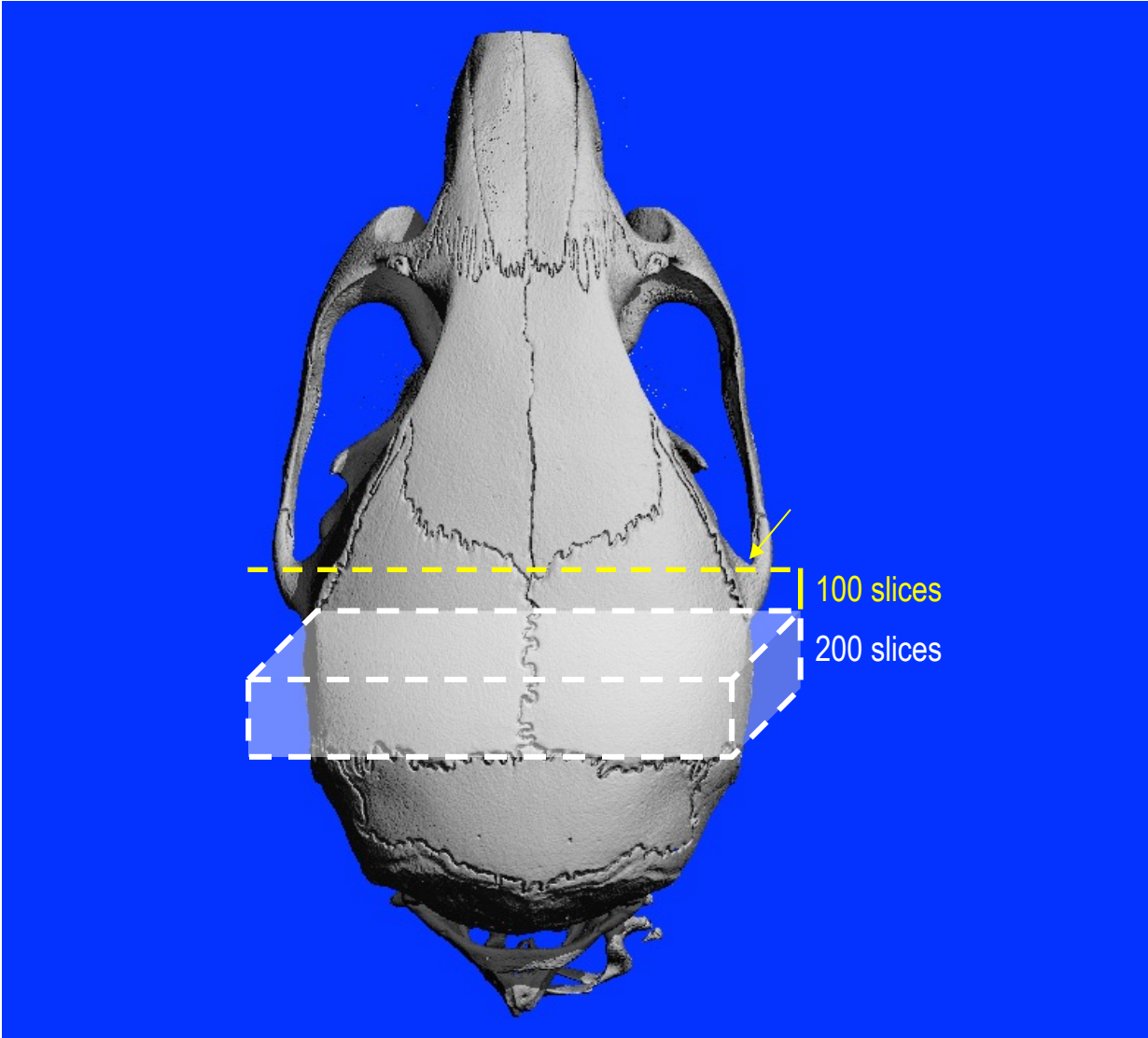

Supplement: SupplementalFigure1_ziae011 [file supplementalfigure1_ziae011.pdf]

## Slide 1
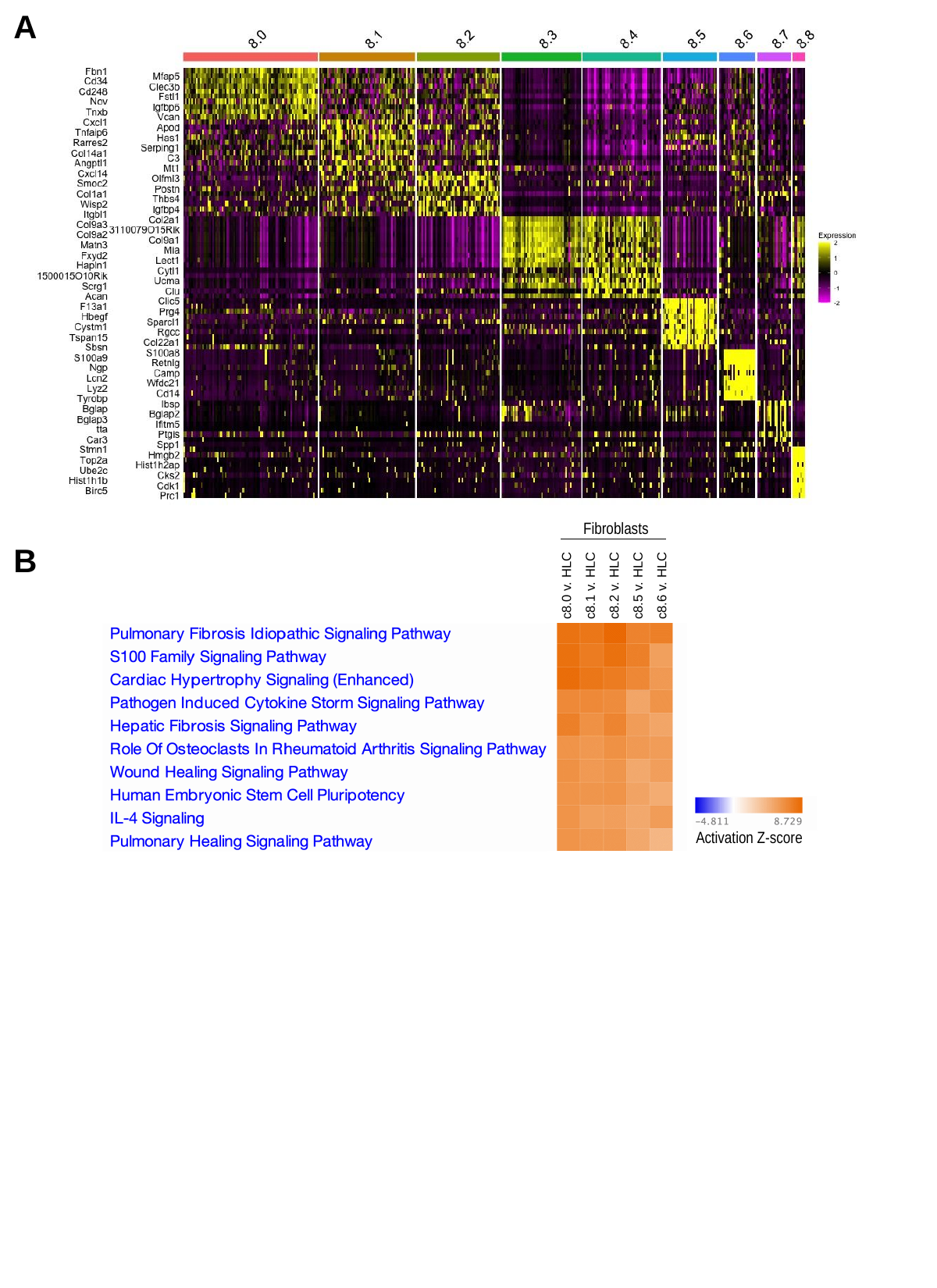

A
Fibroblasts
c8.0 v. HLC
c8.1 v. HLC
c8.2 v. HLC
c8.5 v. HLC
c8.6 v. HLC
Activation Z-score
B

Supplement: SupplementalFigure3-20240112_ziae011 [file supplementalfigure3-20240112_ziae011.pptx]

## Secreted Factors

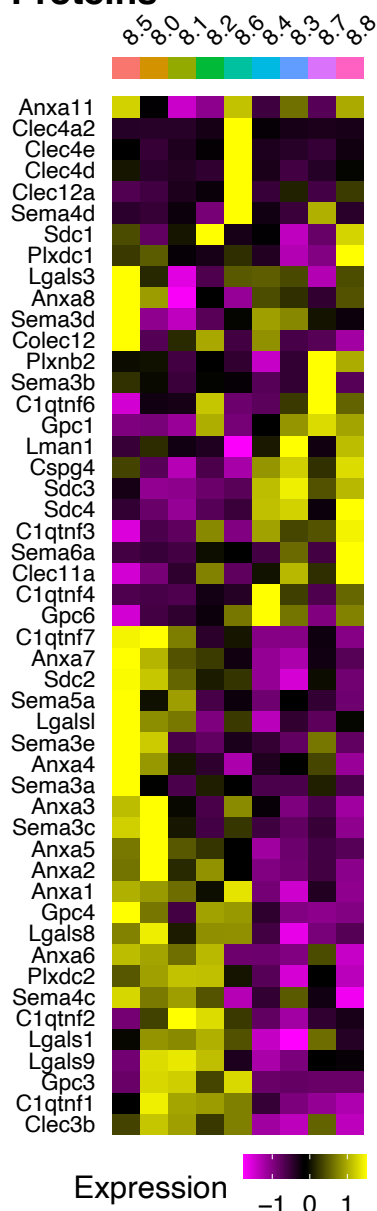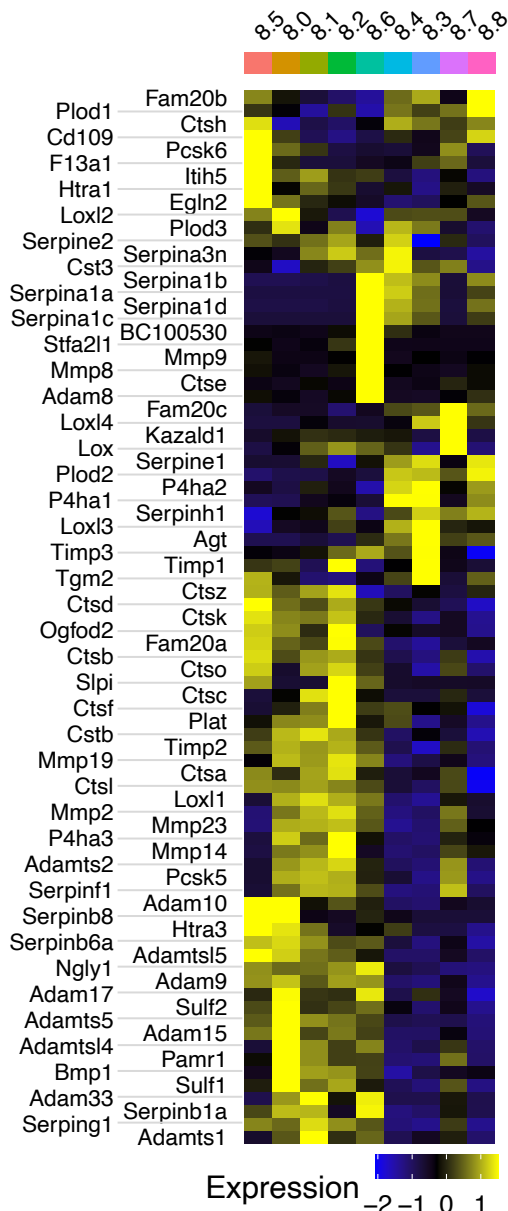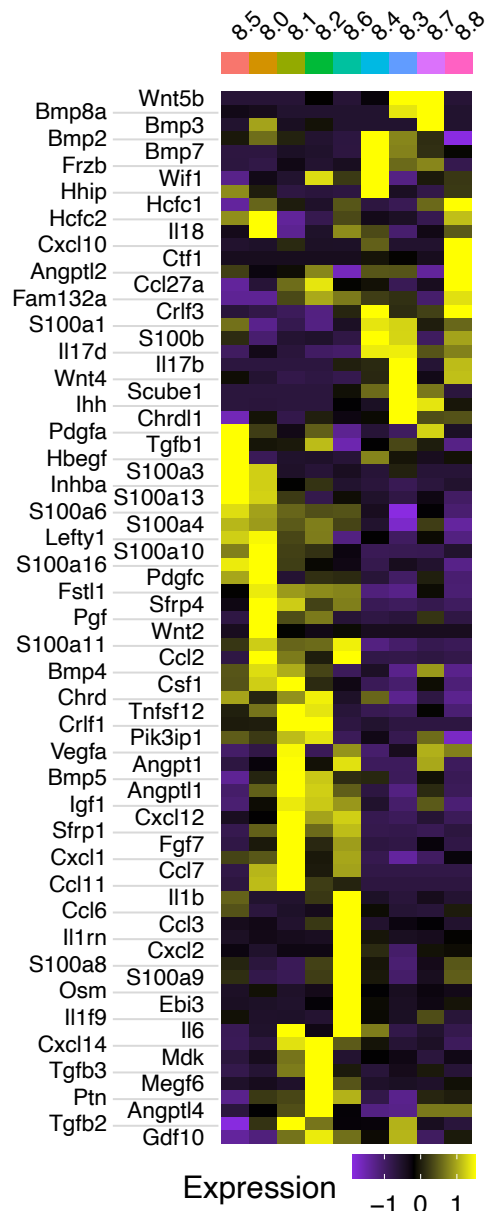

# B

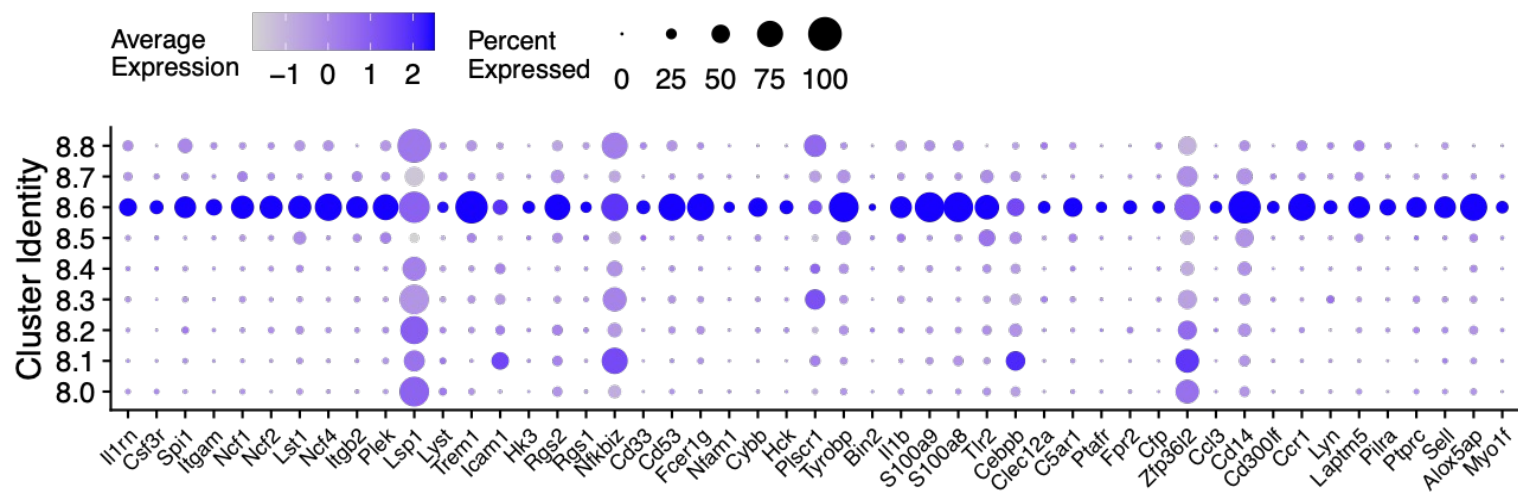

Supplement: SupplementalFigure4-20240112_ziae011 [file supplementalfigure4-20240112_ziae011.pdf]

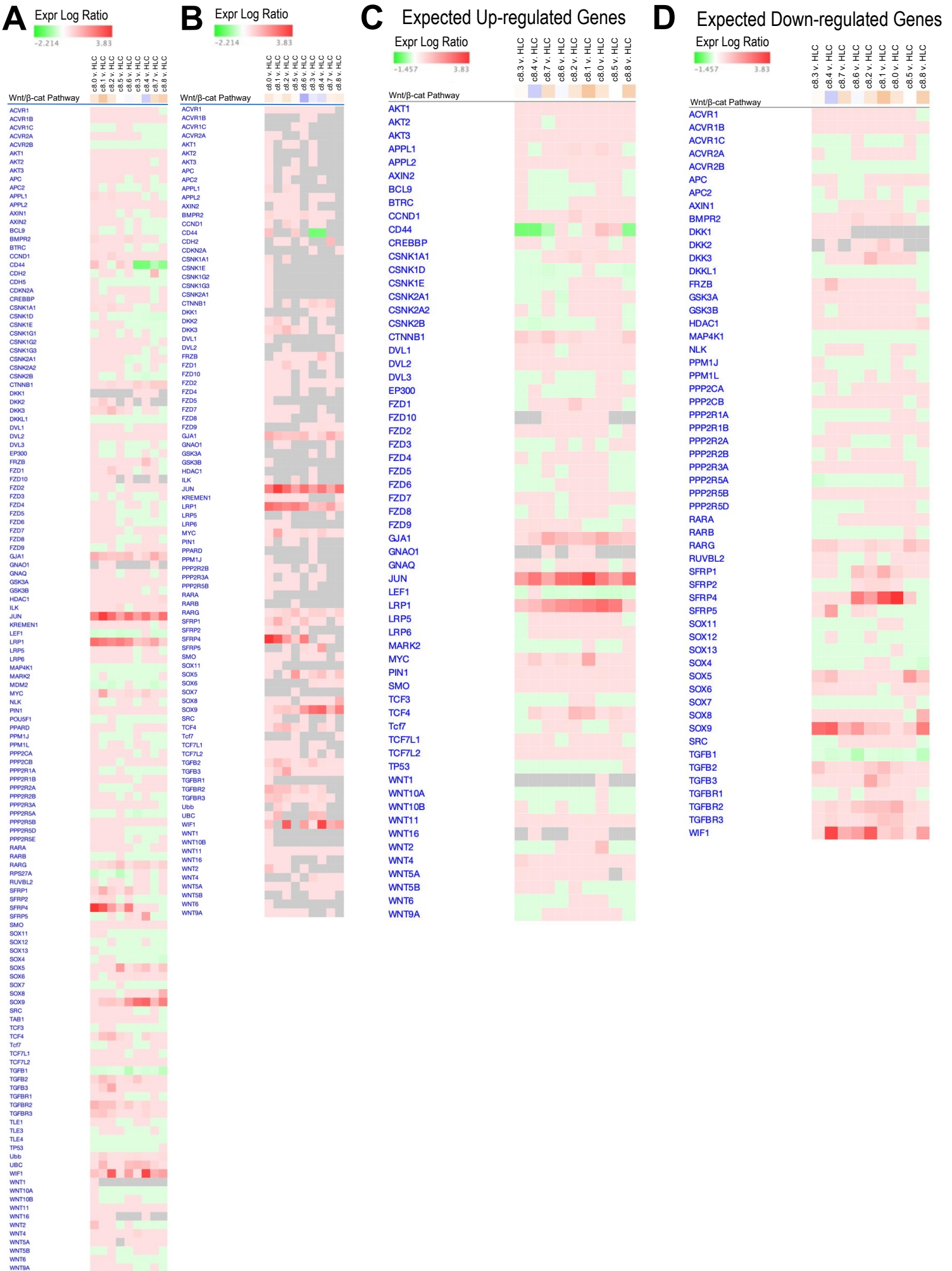

Supplement: SupplementalFigure5-20240112_ziae011 [file supplementalfigure5-20240112_ziae011.pdf]

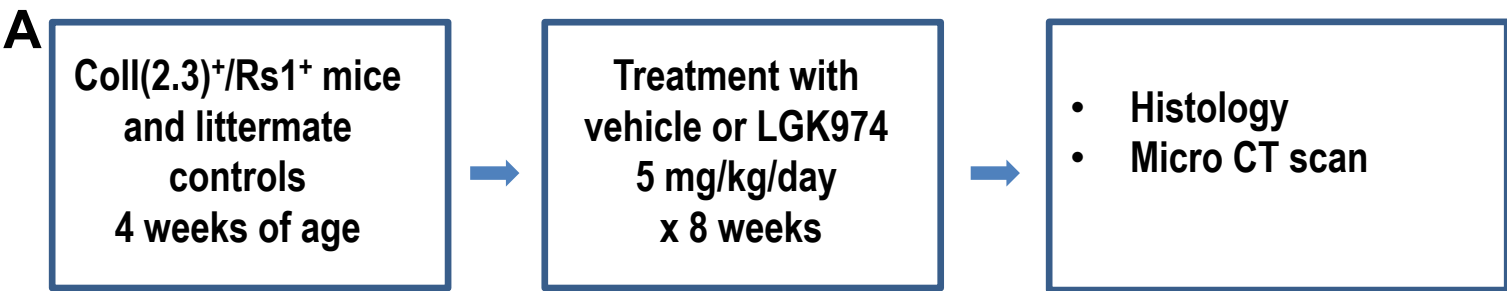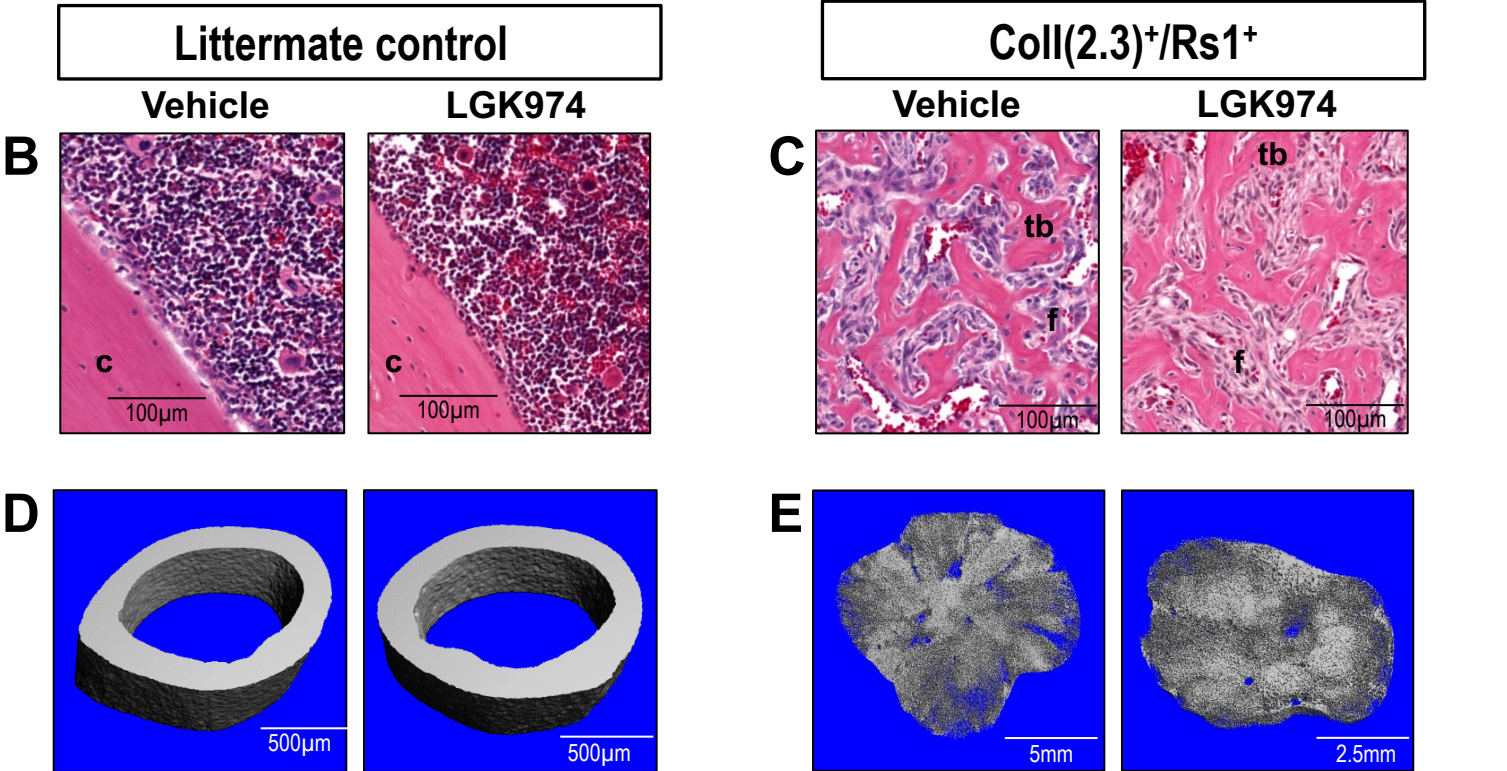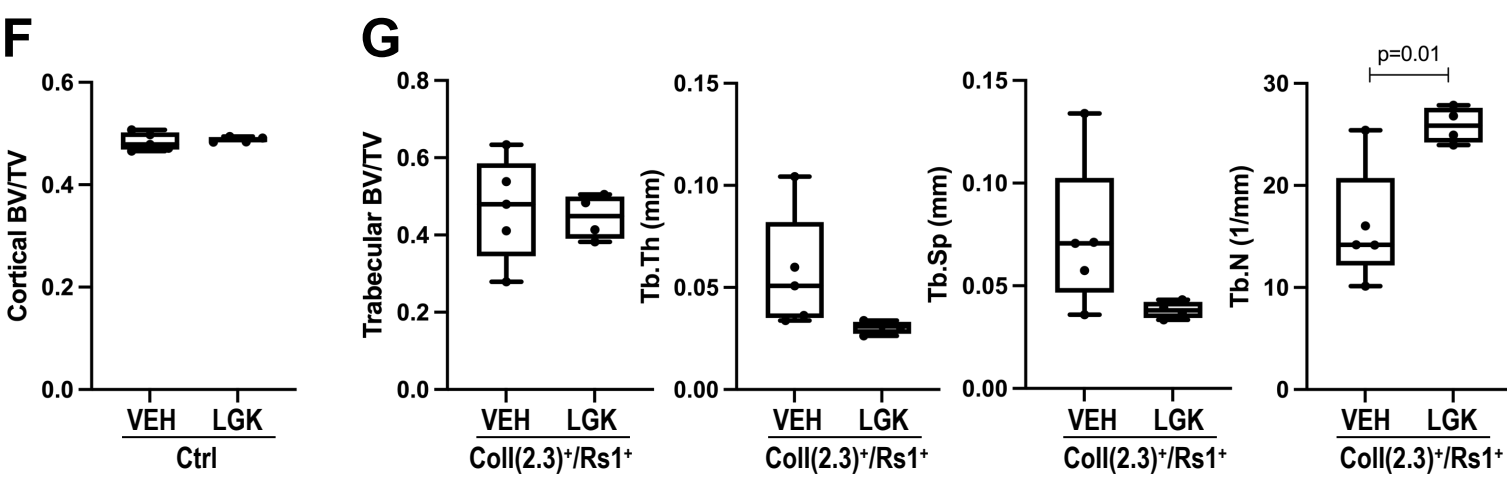

Supplement: SupplementalFigure6-20240112_ziae011 [file supplementalfigure6-20240112_ziae011.pdf]

**A**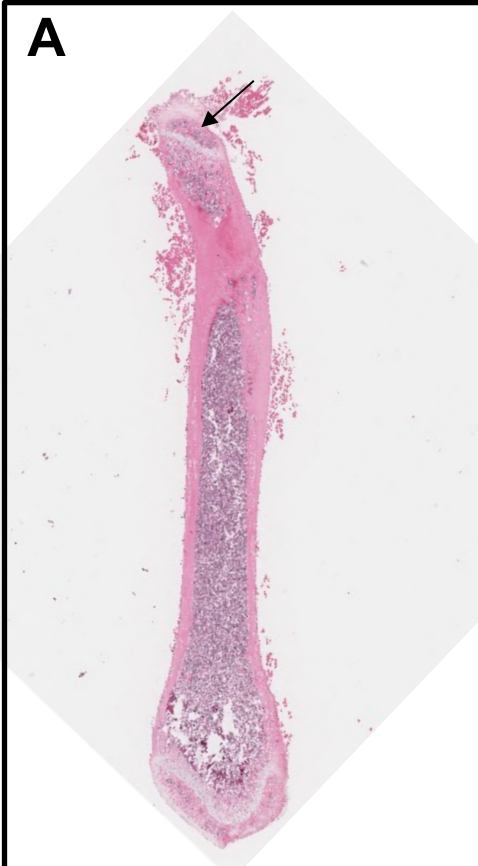

6mm

**B**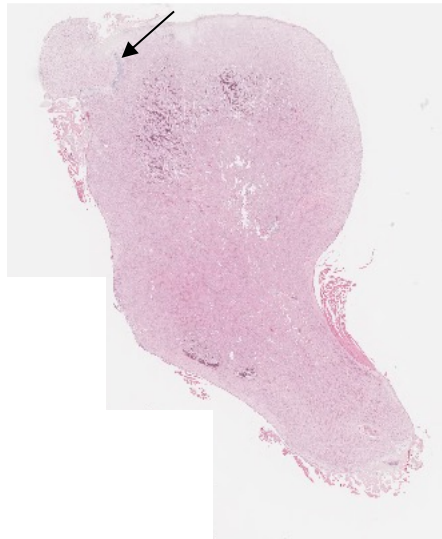

7mm

Supplement: SupplementalFigure7-20240112_ziae011 [file supplementalfigure7-20240112_ziae011.pdf]

**A**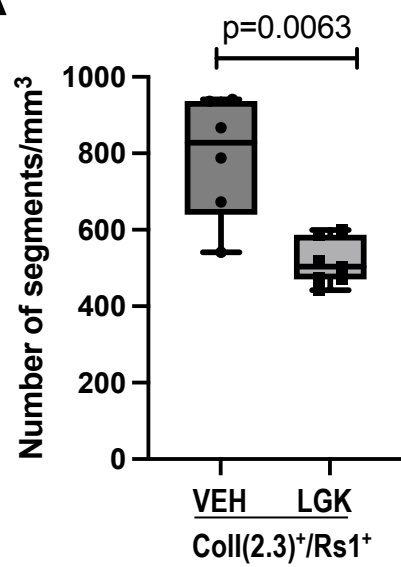**B**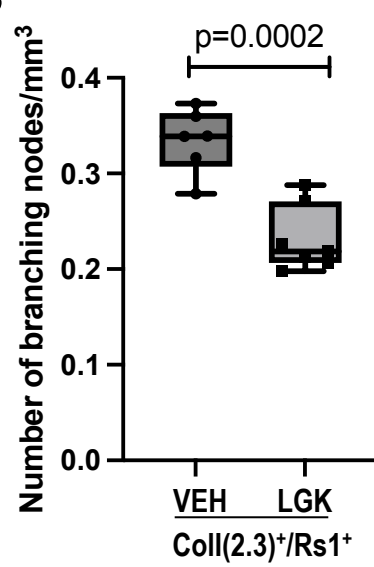

Supplement: SupplementalFigure8-20240112_ziae011 [file supplementalfigure8-20240112_ziae011.pdf]

**A. Osterix (Sp7)**

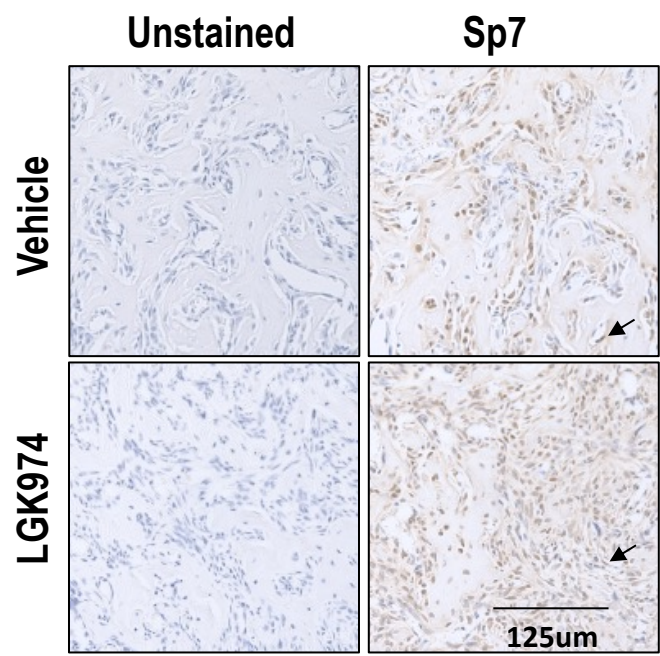

**B. Osteocalcin (OCN)**

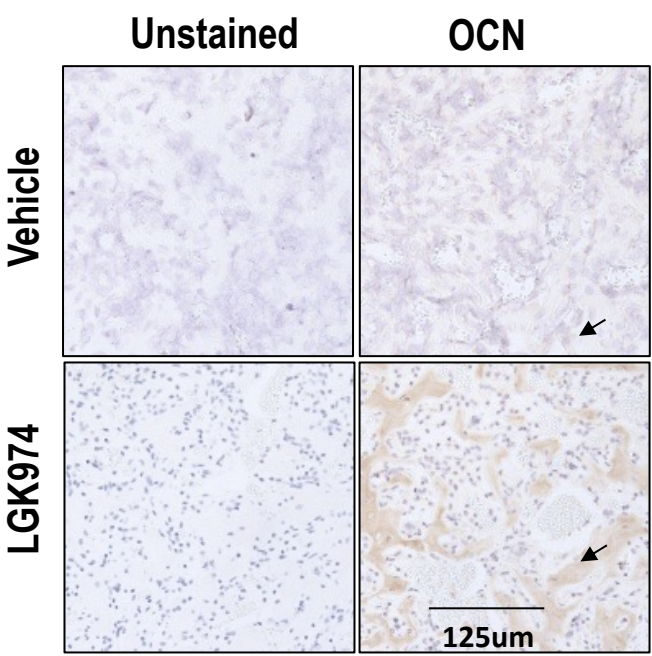

**C. Cathepsin K (CTSK)**

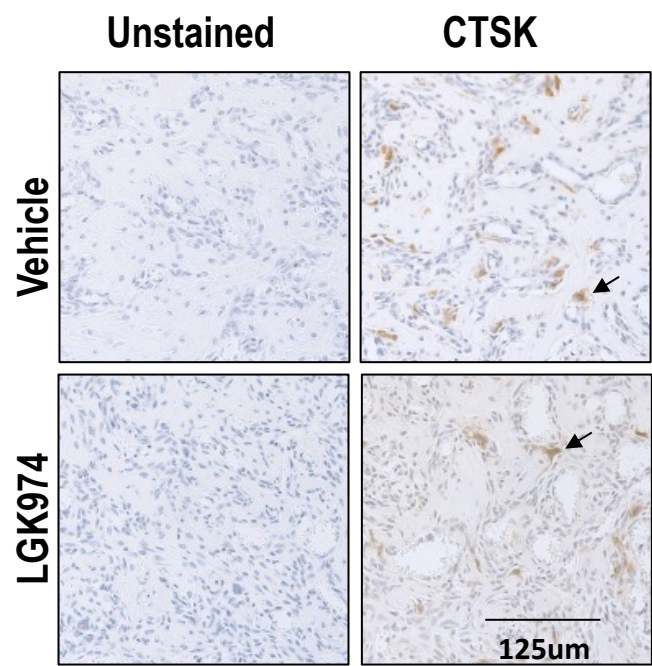

Supplement: SupplementalFigure9-20240112_ziae011 [file supplementalfigure9-20240112_ziae011.pdf]

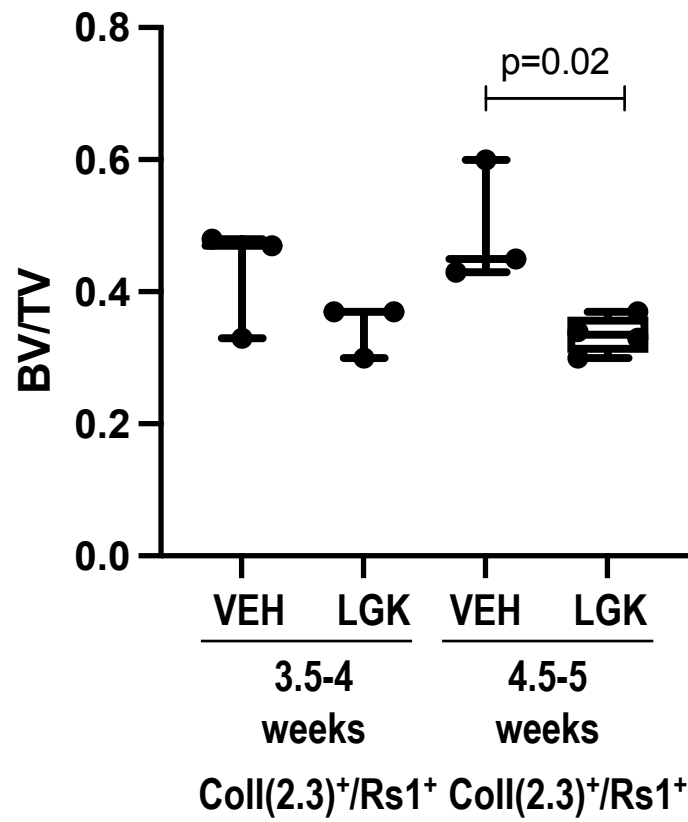

Supplement: SupplementalFigure10-20240112_ziae011 [file supplementalfigure10-20240112_ziae011.pdf]

# Treatment duration

3.5-4 weeks

4.5-5 weeks

**A**

Coll<sup>+/</sup>/Rs1<sup>+</sup> + VEH

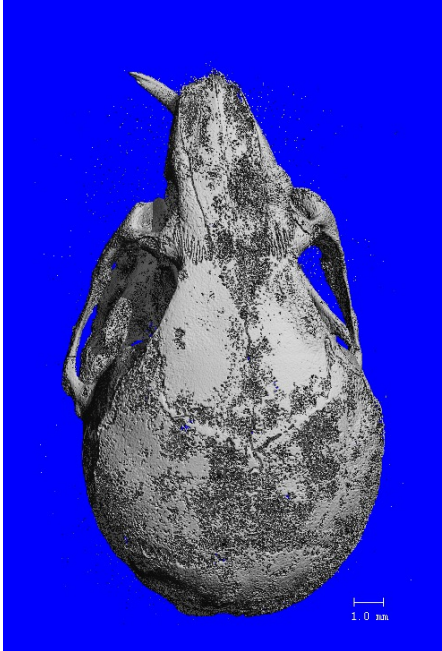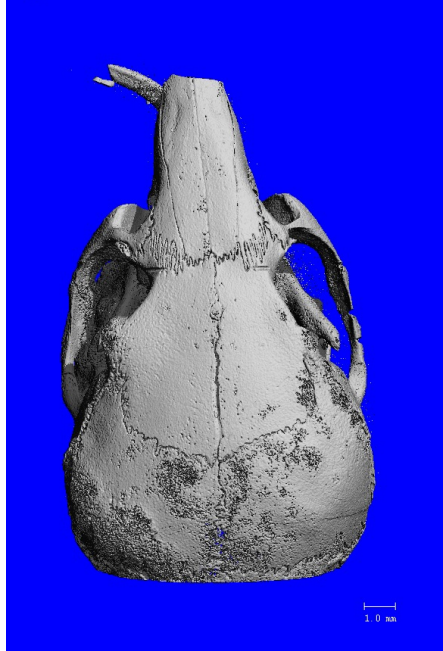

**B**

Coll<sup>+/</sup>/Rs1<sup>+</sup> + LGK

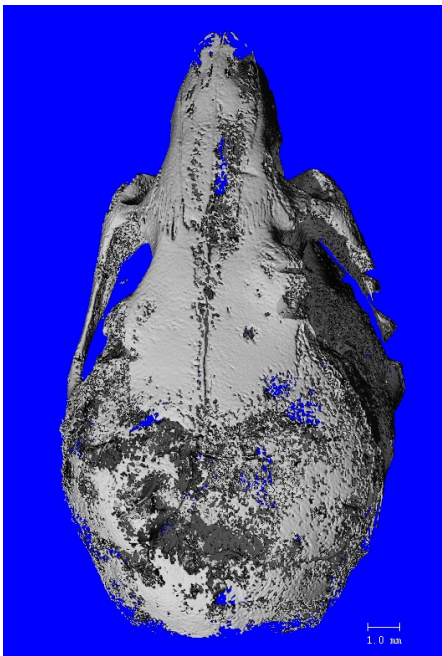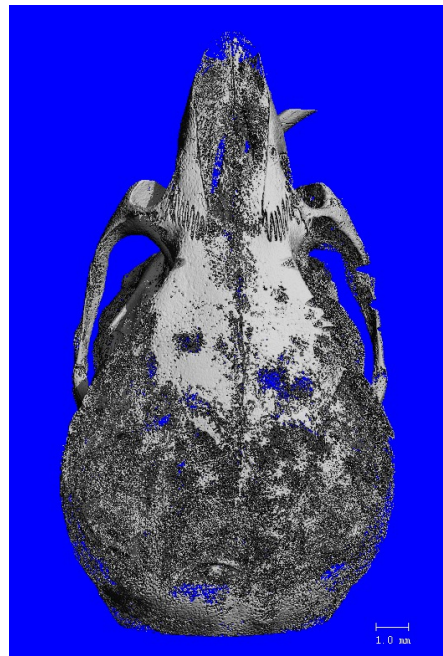

**C**

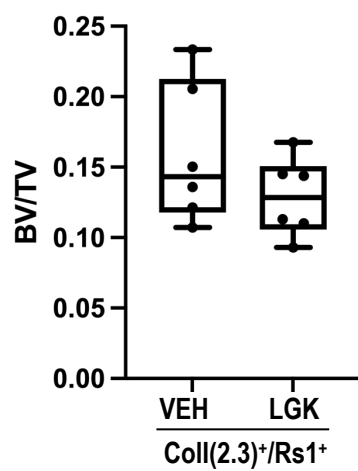

**D**

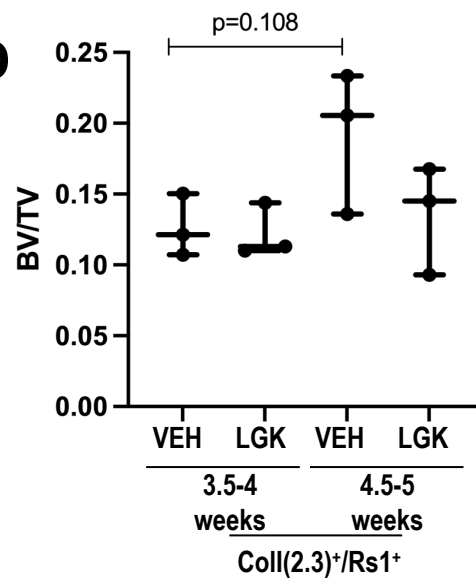

Supplement: SupplementalFigure11-20240112_ziae011 [file supplementalfigure11-20240112_ziae011.pdf]

**A**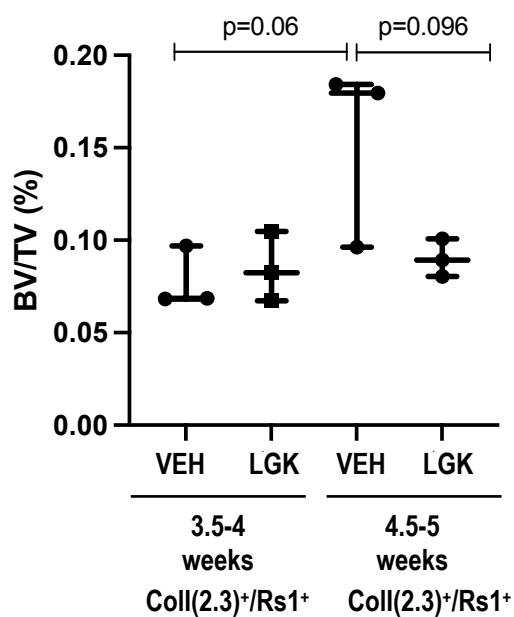**B**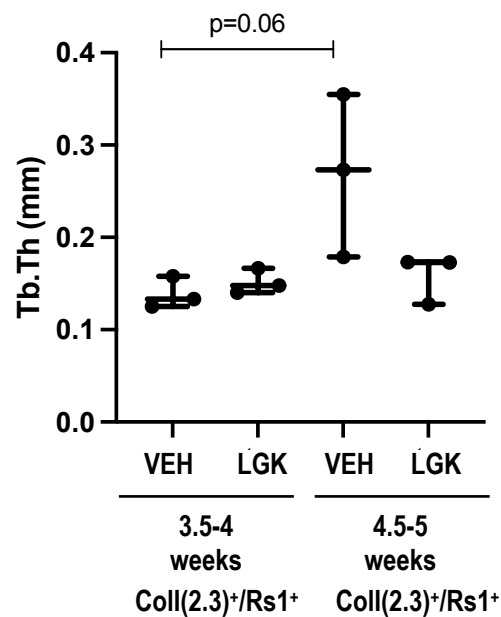**C**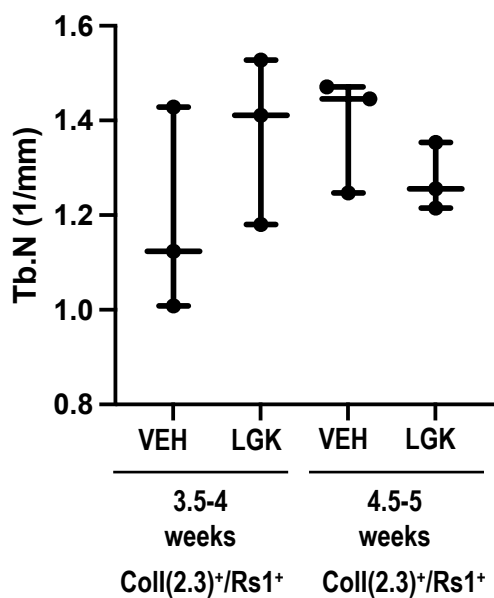**D**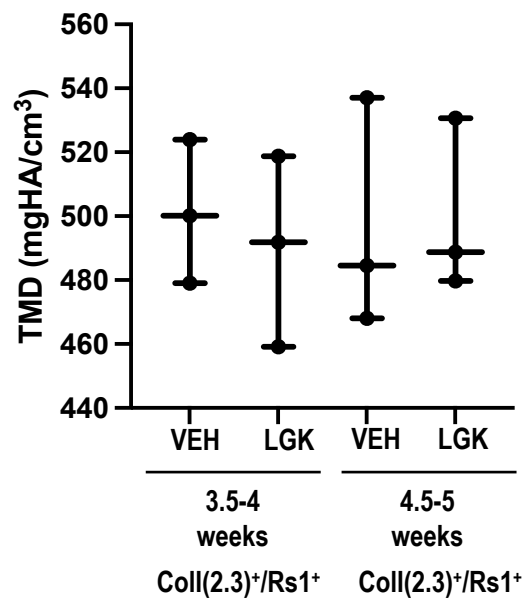**E**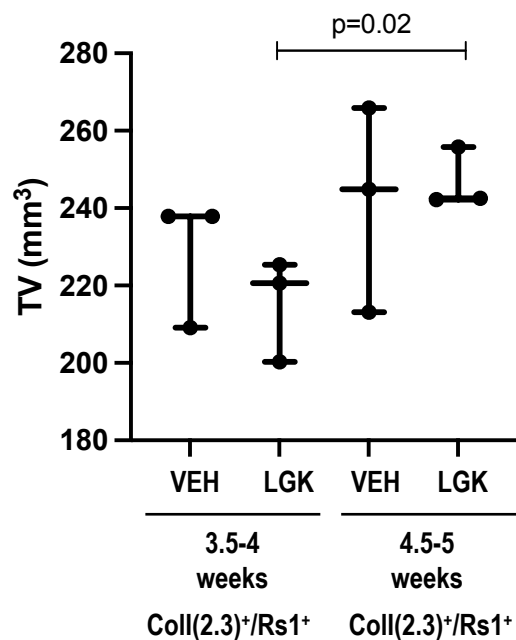**F**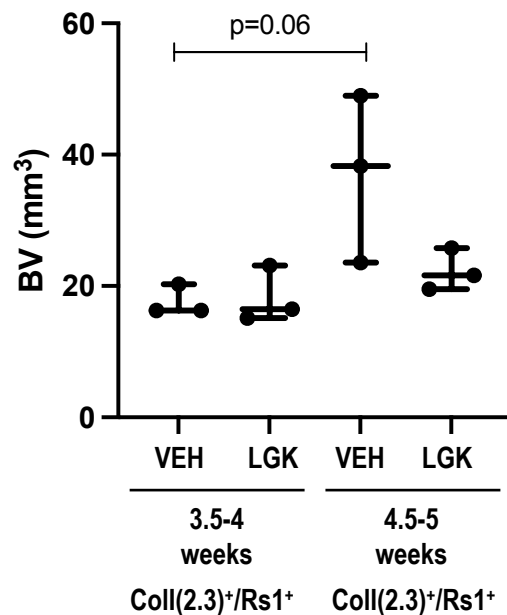

Supplement: SupplementalFigure12-20240112_ziae011 [file supplementalfigure12-20240112_ziae011.pdf]
