# Supplementary material for: Wnt pathway inhibition with the porcupine inhibitor LGK974 decreases trabecular bone but not fibrosis in a murine model with fibrotic bone
Source: JBMR Plus. 2024 Jan 21;8(5):ziae011. doi: 10.1093/jbmrpl/ziae011 (PMC10994528; doi:10.1093/jbmrpl/ziae011)
Supplement: SupplementalFigure2-20240112_ziae011 [file supplementalfigure2-20240112_ziae011.pdf]

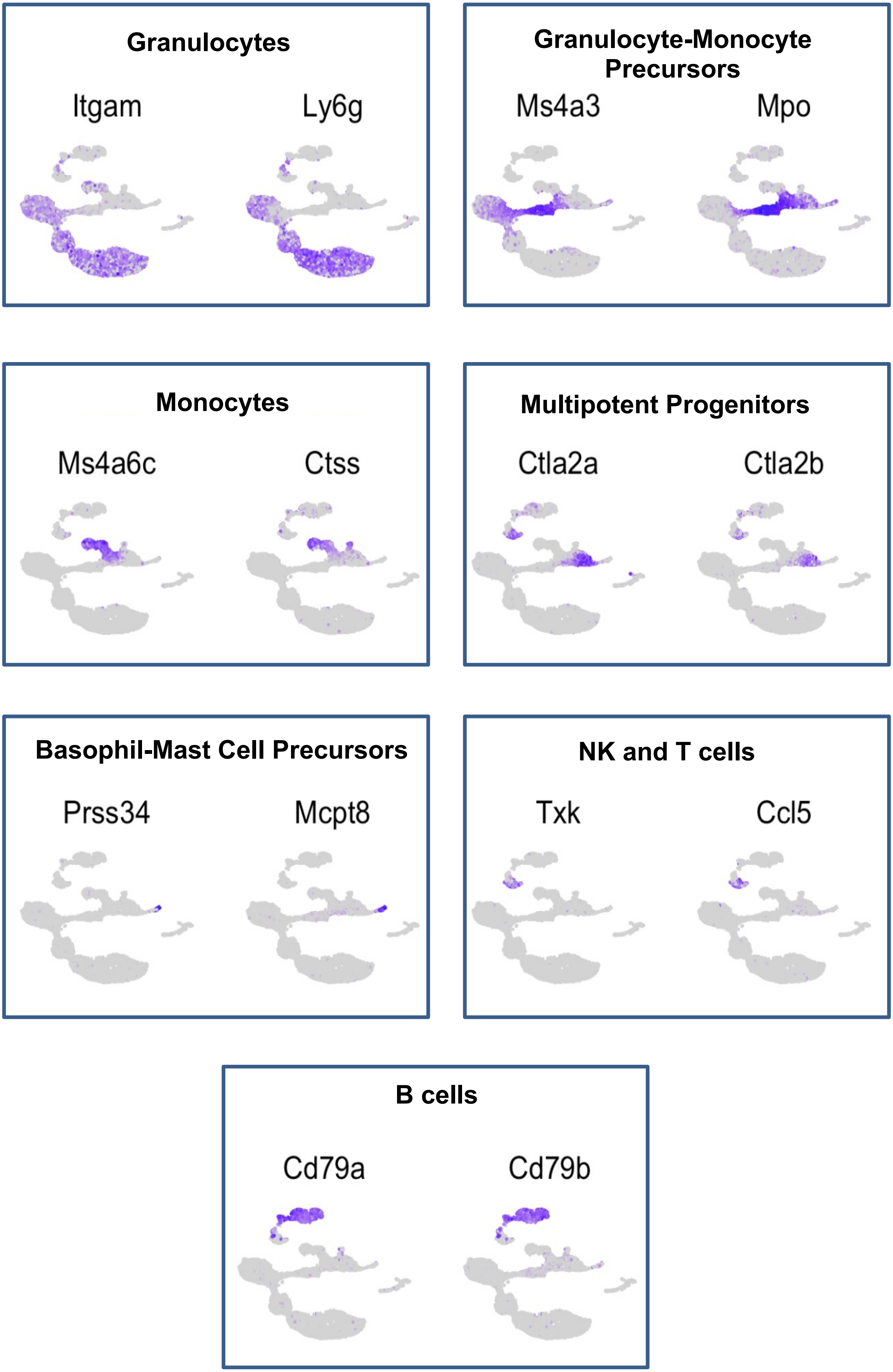
Granulocytes
Itgam
Ly6g
Granulocyte-Monocyte Precursors
Ms4a3
Mpo
Monocytes
Ms4a6c
Ctss
Multipotent Progenitors
Ctla2a
Ctla2b
Basophil-Mast Cell Precursors
Prss34
Mcpt8
NK and T cells
Txk
Ccl5
B cells
Cd79a
Cd79b
