# Supplementary material for: Wnt pathway inhibition with the porcupine inhibitor LGK974 decreases trabecular bone but not fibrosis in a murine model with fibrotic bone
Source: JBMR Plus. 2024 Jan 21;8(5):ziae011. doi: 10.1093/jbmrpl/ziae011 (PMC10994528; doi:10.1093/jbmrpl/ziae011)
Supplement: JBMR_Plus_SupplementalTables_1_2_3_ziae011 [file jbmr_plus_supplementaltables_1_2_3_ziae011.pdf]

Supplemental Table 1	CTRL1		CTRL2		Col1(2.3$^+$)/Rs1$^+$-1		Col1(2.3$^+$)/Rs1$^+$-2	
**Dataset Filtering Steps**	**# of Cells Removed**	**# of Cells Remaining**	**# of Cells Removed**	**# of Cells Remaining**	**# of Cells Removed**	**# of Cells Remaining**	**# of Cells Removed**	**# of Cells Remaining**
1. Number of cells in CellRanger filtered output		7974		6874		7463		6484
2. Remove background mRNA with SoupX R package	0	7974	0	6874	0	7463	0	6484
3. Remove cells with fewer than 200 genes and remove genes expressed in fewer than 3 cells	1840	6134	1313	5561	1018	6445	1100	5384
4. Remove red blood cells by filtering out cells with greater than 100 Hba-a1 gene transcripts	2510	3624	3110	2451	2694	3751	3212	2172
5. Remove low-quality cells by filtering out cells with greater than 5% mitochondrial genes	42	3582	14	2437	31	3720	18	2154
6. Remove probable doublets by filtering out cells with greater than 5500 genes and remove transcriptome size outliers by removing cells with less than 600 genes	14	3568	0	2437	5	3715	1	2153
7. Remove cells identified as 'Erythrocytes' by SingleR	127	**3441**	111	**2326**	153	**3562**	83	**2070**

Supplemental Table 2											
**Cluster**	**0**	**1**	**2**	**3**	**4**	**5**	**6**	**7**	**8**	**9**	**10**
**SingleR Mouse-RNAseq Predicted Cell Type**											
• B cells	0	0	1	2	969	13	240	0	0	4	1
• Dendritic cells	0	0	0	0	0	0	1	0	0	8	5
• Endothelial cells	0	0	0	0	0	0	0	0	15	0	0
• Fibroblasts	0	0	0	0	0	0	2	0	390	0	0
• Granulocytes	2440	1653	1589	1317	141	256	54	21	5	30	40
• Macrophages	0	0	0	0	0	2	1	1	3	0	0
• Monocytes	2	2	0	9	2	757	440	537	3	0	44
• NK cells	0	0	0	1	0	0	44	0	0	169	2
• T cells	1	2	0	0	0	2	101	0	0	76	1

Supplemental Table 3										
	Subcluster Number									
	**8.0**	**8.1**	**8.2**	**8.3**	**8.4**	**8.5**	**8.6**	**8.7**	**8.8**	**Total**
**All cells (CTRL+Coll(2.3)+/Rs1^{+})**	84	60	52	50	49	34	23	21	8	381
**CTRL1**	1	1	1	15	12	4	3	2	2	41
**CTRL2**	1	0	0	3	3	1	0	0	1	9
• **CTRL Total**	2	1	1	18	15	5	3	2	3	50
**Coll(2.3)+/Rs1^{+}-1**	62	36	35	25	29	24	16	14	4	245
**Coll(2.3)+/Rs1^{+}-2**	20	23	16	7	5	5	4	5	1	86
• **Coll(2.3)+/Rs1^{+} Total**	82	59	51	32	34	29	20	19	5	331
