# Supplementary material for: Wnt pathway inhibition with the porcupine inhibitor LGK974 decreases trabecular bone but not fibrosis in a murine model with fibrotic bone
Source: JBMR Plus. 2024 Jan 21;8(5):ziae011. doi: 10.1093/jbmrpl/ziae011 (PMC10994528; doi:10.1093/jbmrpl/ziae011)
Supplement: JBMR-Plus-SupplementalFigures_Tables_Legend-20240217_ziae011 [file jbmr-plus-supplementalfigures_tables_legend-20240217_ziae011.pdf]

Supplemental Figures and Tables:

Supplemental Figure 1. Defining the region of interest (ROI) for craniofacial trabecular bone assessment. A 200-slice ROI was manually contoured for trabecular bone assessment. The medial aspect of the cranial-most portion of the posterior attachment of the zygomatic arch was used as the reference landmark (yellow arrow pointing to the attachment site; yellow dotted line denoting the cross-sectional start point), and the 200-slice ROI (dotted white box) started 100 slices caudal to this landmark.

Supplemental Figure 2. Cluster identification using known cell markers corresponds with results from SingleR. Known markers of granulocytes, monocytes, erythrocytes, NK cells, and B cells were used to validate the cell types of each cluster in all four samples combined. These results correspond with the results from SingleR.

Supplemental Figure 3. Stromal cell subcluster heatmaps and pathway analysis

(A) Heatmap of the top 10 differentially expressed genes in each subcluster of cluster 8 (c8.0-8.8) versus all other subclusters in cluster 8.

(B) Ingenuity Pathway Analysis (IPA) showing the top 10 pathways based on activation Z-scores in the fibroblastic subclusters of cluster 8 versus the non-stromal cell lineages. Pathway analysis included all genes that were expressed at a log fold change greater than 0.25 and an FDR <0.05.

Supplemental Figure 4. Matrisome gene expression heatmaps

(A) Heatmap depiction of the matrisome-associated genes (ECM-affiliated proteins, ECM regulators and secreted factors) expressed in the stromal cell subclusters (c8.0-8.8). The heatmap color blocks depict average gene expression for a given gene across each cluster.

(B) Dotplot representation of the monocyte gene markers in c8.6 compared to the remaining subclusters. These markers are based on PanglaoDB Augmented 2021 gene lists of monocyte markers[1].

Supplemental Figure 5. Wnt/β-catenin pathway gene regulation

(A) Alphabetical list of genes in the Wnt/β-catenin signaling network, independent of statistical significance, that are up- or down-regulated in subclusters c8.0-8.8 compared to the remaining non-stromal hematopoietic lineage cell (HLC) populations. This analysis was performed using the 194 genes that are associated with the Wnt signaling pathway in IPA.

(B) Alphabetical list of genes in the Wnt/β-catenin signaling network with an FDR <0.05 (statistically significant) that are up- or down-regulated in subclusters c8.0-8.8 compared to the remaining non-

stromal hematopoietic lineage cell (HLC) populations. This analysis was performed using the 194 genes that are associated with the Wnt signaling pathway in IPA.

(C) Alphabetical list of all genes that are expected to be ***up***-regulated when the Wnt/β-catenin signaling pathway is activated in subcluster 8 compared to the remaining non-stromal HLC populations, independent of statistical significance. This analysis was performed using the 194 genes that are associated with the Wnt signaling pathway in IPA.

(D) Alphabetical list of all genes that are expected to be ***down***-regulated when the Wnt/β-catenin signaling pathway is activated in subcluster 8 compared to the remaining non-stromal HLC populations, independent of statistical significance. This analysis was performed using the 194 genes that are associated with the Wnt signaling pathway in IPA.

Supplemental Figure 6. Treatment with low-dose LGK974 (5mg/kg/day) leads to minimal changes in fibrous dysplastic-like trabecular bone in ColI(2.3)$^+$/Rs1$^+$ mice

(A) Experimental flow of 4-week-old ColI(2.3)$^+$/Rs1$^+$ and control mice treated with vehicle or LGK974 (5 mg/kg/day) via oral gavage for 8 weeks.

(B) H&E staining of control longitudinal-sectioned femurs (c=cortical bone).

(C) H&E staining of longitudinal-sectioned ColI(2.3)$^+$/Rs1$^+$ femurs (tb=trabecular bone; f=fibrous tissue).

(D) 3D reconstruction of microCT images of control mid-femoral cross-sections at 5um resolution, which is comprised primarily of cortical bone.

(E) 3D reconstruction of microCT images of ColI(2.3)$^+$/Rs1$^+$ mid-femoral cross-sections at 5um resolution, with trabecular bone visible in the bone marrow space.

(F) MicroCT cortical analyses of control mid-femurs show no significant difference in BV/TV after LGK974 treatment. n=5 control with vehicle, n=4 control with LGK974. $p<0.05$ denotes significance.

(G) MicroCT trabecular analyses of ColI(2.3)$^+$/Rs1$^+$ mice show no significant differences in BV/TV, trabecular thickness (Tb.Th), or trabecular separation (Tb.Sp) after LGK974 treatment. A significant increase in trabecular number (Tb.N) ($p=0.01$) was seen. n=5 ColI(2.3)$^+$/Rs1$^+$ with vehicle, n=4 ColI(2.3)$^+$/Rs1$^+$ with LGK974.

Ctrl=control; VEH=vehicle; LGK=LGK974; TV=tissue volume; BV=bone volume; BV/TV=bone volume-to-tissue volume ratio or bone volume fraction; Tb.Th=trabecular thickness, Tb.Sp = trabecular separation; Tb.N=trabecular number.

Supplemental Figure 7. Sagittal histological sections of control and Col1(2.3)$^+$/Rs1$^+$ mouse femurs at 9 weeks of age

(A) Control bone, 9 weeks of age. Black arrow depicts the growth plate.

(B) ColI(2.3)$^{+}$/Rs1^{+} bone at 9 weeks of age showing diffuse, dense trabecularization throughout the bone with obliteration of typical anatomical sites. Black arrow depicts the growth plate.

Supplemental Figure 8. Additional trabecular assessments of ColI(2.3)$^{+}$/Rs1^{+} mice treated with high-dose LGK974

(A) Assessment of the number of mineralized bone segments (p=0.0063)

(B) Assessment of the number of branching nodes (p=0.0002)

Supplemental Figure 9. Immunohistochemistry (IHC) shows persistent expression of osterix (Sp7), osteocalcin (OCN), and cathepsin K (CTSK) in ColI(2.3)$^{+}$/Rs1^{+} mice.

(A) IHC Sp7 staining: Sp7 positive cells are present after LGK974 treatment (black arrow).

(B) IHC Osteocalcin: osteocalcin is present in the matrix after LGK974 treatment (black arrow).

(C) IHC Cathepsin K: osteoclasts are present after LGK974 treatment (black arrow).

Supplemental Figure 10. Subgroup analysis of ColI(2.3)$^{+}$/Rs1^{+} mice by high-dose LGK974 treatment duration. Sub-group analysis by treatment duration shows that ColI(2.3)$^{+}$/Rs1^{+} mice treated for 4.5-5 weeks with LGK974 30mg/kg/day have significant thinning of fibrous dysplastic trabecular bone in the femur (p=0.02) whereas those treated for 3.5-4 weeks did not reach statistical significance (p=0.2071). Mouse numbers for 3.5-4 weeks: ColI(2.3)$^{+}$/Rs1^{+} vehicle-treated, n=3, LGK974-treated, n=3; 4.5-5 weeks: ColI(2.3)$^{+}$/Rs1^{+} vehicle-treated, n=3, LGK974-treated, n=4.

Supplemental Figure 11. 3D images and microstructural assessment of whole skulls from ColI(2.3)$^{+}$/Rs1^{+} mice treated with high-dose LGK974 (30 mg/kg/day)

(A) Representative images of whole skulls from ColI(2.3)$^{+}$/Rs1^{+} mice treated with vehicle or **(B)** LGK974. Images were chosen based on the microstructural trabecular quantification median. The sample size for vehicle-treated ColI(2.3)$^{+}$/Rs1^{+} mice at 3.5-4 weeks is n=3 and at 4.5-5 weeks is n=3. The sample size for LGK974-treated ColI(2.3)$^{+}$/Rs1^{+} mice at 3.5-4 weeks is n=3 and at 4.5-5 weeks is n=3. Scale bars=1 mm.

(C) MicroCT analyses of the whole skull of ColI(2.3)$^{+}$/Rs1^{+} mice showed no significant difference in BV/TV between vehicle and LGK-treated mice over the treatment period.

(D) A non-significant increase in BV/TV (p=0.108) was detected in vehicle ColI(2.3)$^{+}$/Rs1^{+} mice treated for 3.5-4 weeks versus 4.5-5 weeks. No significant differences were detected between vehicle and LGK-treated ColI(2.3)$^{+}$/Rs1^{+} mice over any time period.

Ctrl=control; VEH=vehicle; LGK=LGK974; TV=tissue volume; BV=bone volume; BV/TV=bone volume-to-tissue volume ratio

Supplemental Figure 12. Microstructural assessment of the 200-slice ROI in ColI(2.3)$^+$/Rs1$^+$ mice by duration of treatment (3.5-4 weeks versus 4.5-5 weeks) with high-dose (30mg/kg/day) LGK974

(A) There is a trend towards an increase in BV/TV between vehicle-treated ColI(2.3)$^+$/Rs1$^+$ mice at 3.5-4 weeks and 4.5-5 weeks (p = 0.06), highlighting the natural progression of FD lesions over time. There is no difference between LGK974-treated ColI(2.3)$^+$/Rs1$^+$ mice at 3.5-4 weeks and 4.5-5 weeks, highlighting the possible decrease in FD lesion progression with a longer duration of treatment.

(B) There is a trend towards an increase in Tb.Th between vehicle-treated ColI(2.3)$^+$/Rs1$^+$ mice at 3.5-4 weeks and 4.5-5 weeks (p=0.06), highlighting the natural progression of FD lesions over time. There is no difference in Tb.Th between LGK974-treated ColI(2.3)$^+$/Rs1$^+$ mice at 3.5-4 weeks and 4.5-5 weeks.

(C) There are no changes in Tb.N between vehicle-treated or LGK974-treated ColI(2.3)$^+$/Rs1$^+$ mice at 3.5-4 weeks and 4.5-5 weeks.

(D) There are no changes in TMD between vehicle-treated or LGK974-treated ColI(2.3)$^+$/Rs1$^+$ mice at 3.5-4 weeks and 4.5-5 weeks.

(E) There is an increase in TV between LGK974-treated ColI(2.3)$^+$/Rs1$^+$ mice at 3.5-4 weeks and 4.5-5 weeks. (p=0.02).

(F) There is a trend towards an increase in BV between vehicle-treated ColI(2.3)$^+$/Rs1$^+$ mice at 3.5-4 weeks and 4.5-5 weeks (p=0.06).

Ctrl=control; VEH=vehicle; LGK=LGK974; TV=tissue volume; BV=bone volume; BV/TV=bone volume-to-tissue volume ratio or bone volume fraction; Tb.N=trabecular number; Tb.Th=trabecular thickness; TMD=tissue mineral density

SUPPLEMENTAL TABLES:

Supplemental Table 1. Dataset filtering and Quality Control (QC) steps

The filtering steps, including the number of cells that were filtered out, as well as the number of remaining cells after each filtering step, are shown here. These procedures were done in Seurat. CTRL=control

Supplemental Table 2. SingleR cell type prediction. The number of cells in each cluster that correspond to a cell type predicted by SingleR.

Supplemental Table 3. Cell numbers after subclustering of cluster 8

The number of cells in each subcluster of cluster 8, split by sample.

CTRL=control

REFERENCES IN SUPPLEMENTAL FIGURES:

1. Franzen O, Gan LM, Bjorkegren JLM. PanglaoDB: a web server for exploration of mouse and human single-cell RNA sequencing data. *Database (Oxford).* 2019;2019.
