# Supplementary material for: Wnt pathway inhibition with the porcupine inhibitor LGK974 decreases trabecular bone but not fibrosis in a murine model with fibrotic bone
Source: JBMR Plus. 2024 Jan 21;8(5):ziae011. doi: 10.1093/jbmrpl/ziae011 (PMC10994528; doi:10.1093/jbmrpl/ziae011)
Supplement: JBMR-Plus-SupplementalFigures-20231221_ziae011 [file jbmr-plus-supplementalfigures-20231221_ziae011.pdf]

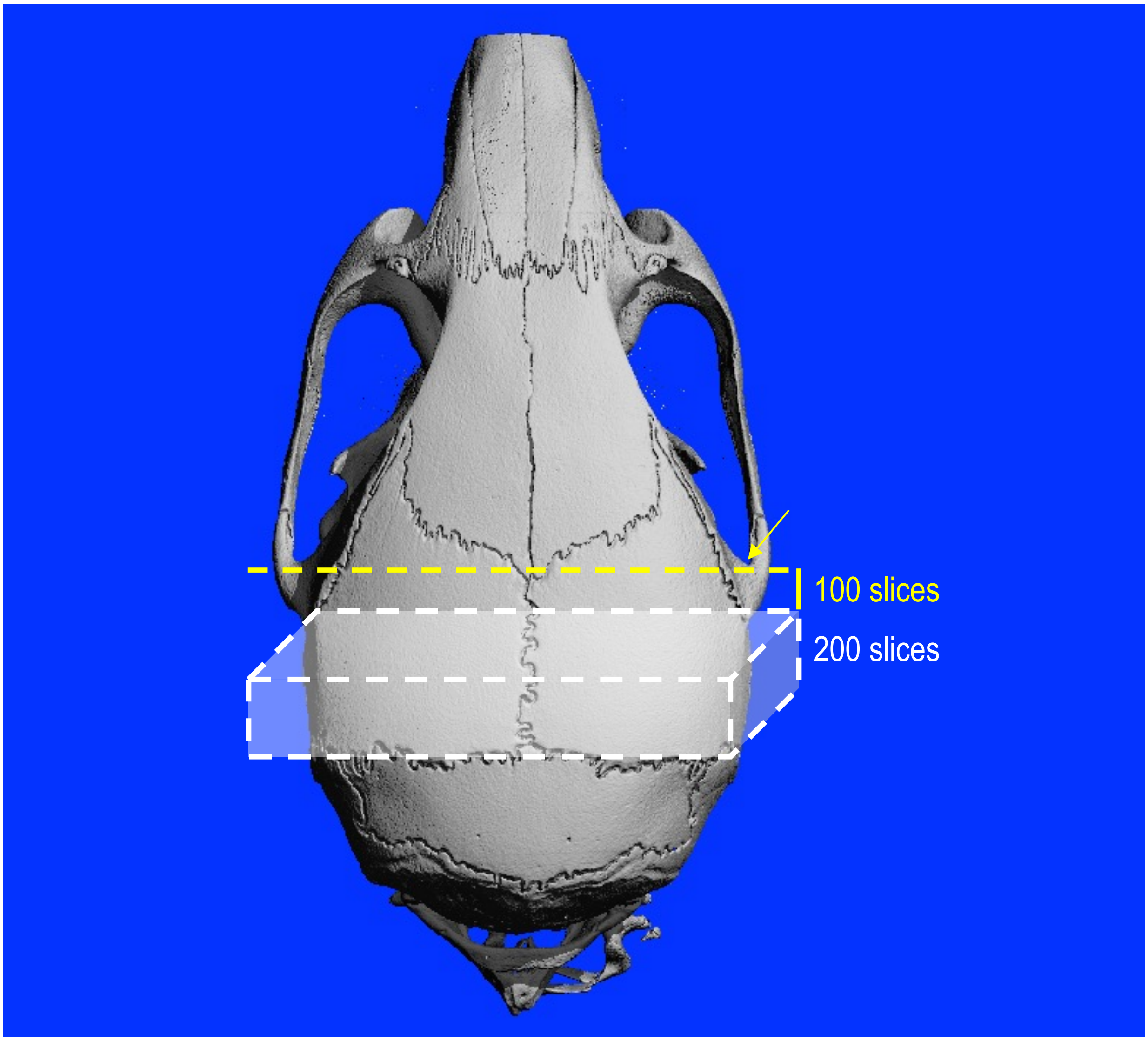
100 slices
200 slices

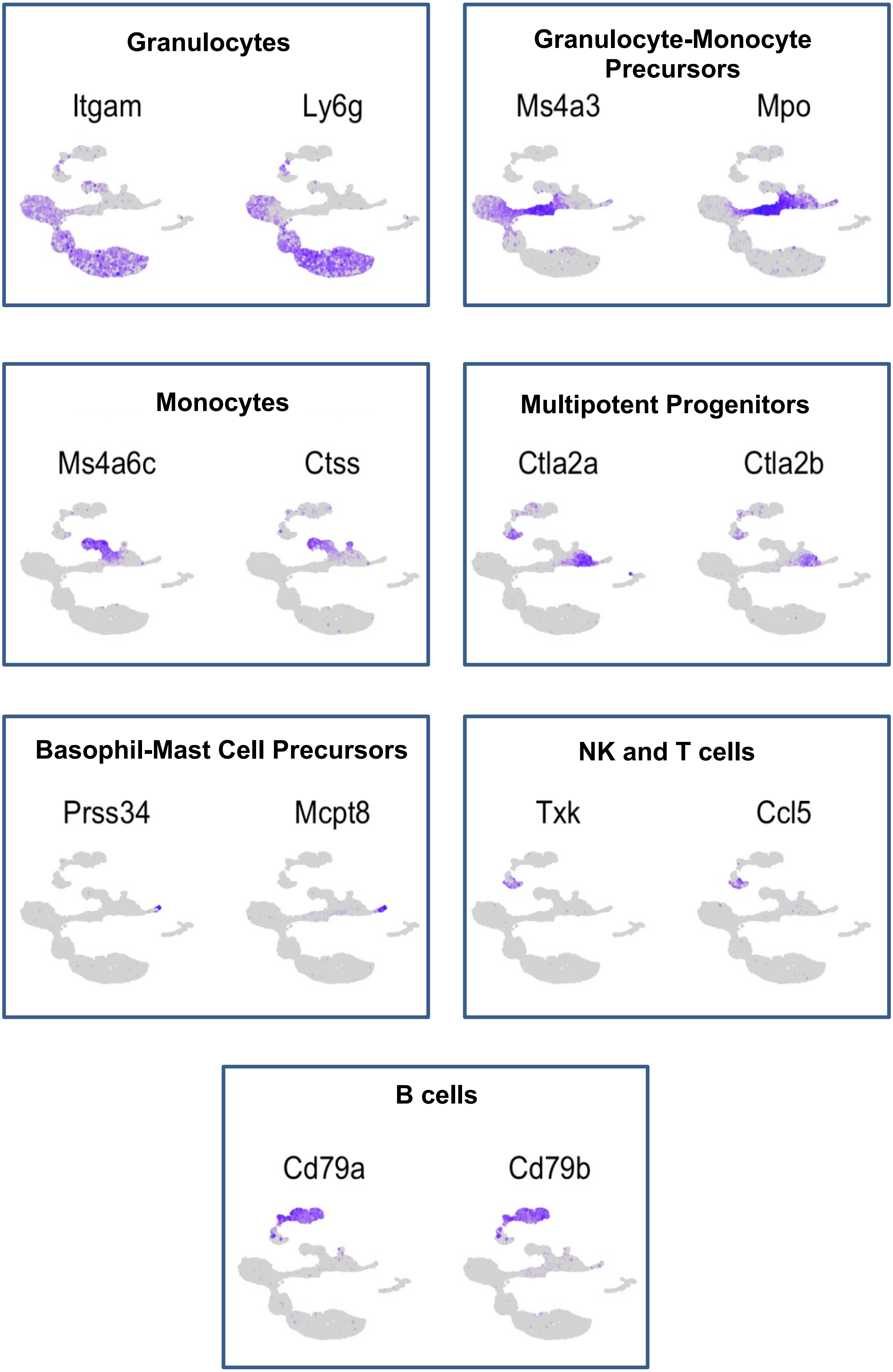
Granulocytes
Itgam
Ly6g
Granulocyte-Monocyte Precursors
Ms4a3
Mpo
Monocytes
Ms4a6c
Ctss
Multipotent Progenitors
Ctla2a
Ctla2b
Basophil-Mast Cell Precursors
Prss34
Mcpt8
NK and T cells
Txk
Ccl5
B cells
Cd79a
Cd79b

SFig 2

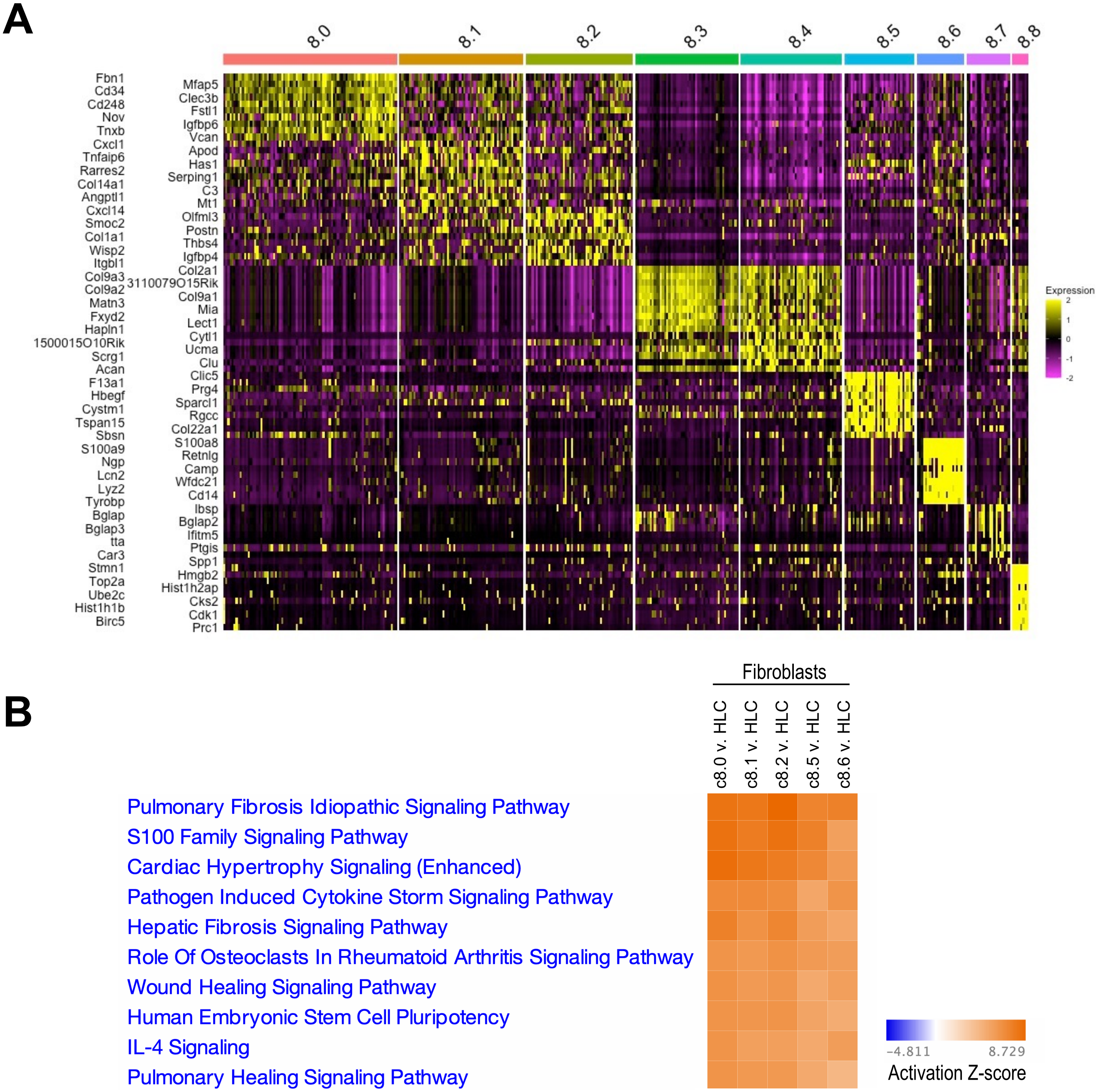
A
8.0
8.1
8.2
8.3
8.4
8.5
8.6
8.7
8.8
Fbn1
Cd34
Cd248
Nov
Tnxb
Cxcl1
Tnfaip6
Rarres2
Col14a1
Angptl1
Cxcl14
Smoc2
Col1a1
Wisp2
Itgbl1
Col9a3
Col9a2
Matn3
Fxyd2
Hapln1
1500015O10Rik
Scrg1
Acan
F13a1
Hbegf
Cystm1
Tspan15
Sbsn
S100a9
Ngp
Lcn2
Lyz2
Tyrobp
Bglap
Bglap3
tta
Car3
Stmn1
Top2a
Ube2c
Hist1h1b
Birc5
Mfap5
Clec3b
Fstl1
Igfbp6
Vcan
Apod
Has1
Serping1
C3
Mt1
Olfml3
Postn
Thbs4
Igfbp4
Col2a1
3110079O15Rik
Col9a1
Mia
Lect1
Cytl1
Ucma
Clu
Clic5
Prg4
Sparcl1
Rgcc
Col22a1
S100a8
Retnlg
Camp
Wfdc21
Cd14
Ibsp
Bglap2
Ifitm5
Ptgis
Spp1
Hmgb2
Hist1h2ap
Cks2
Cdk1
Prc1
Expression
2
1
0
-1
-2
B
Fibroblasts
c8.0 v. HLC
c8.1 v. HLC
c8.2 v. HLC
c8.5 v. HLC
c8.6 v. HLC
Pulmonary Fibrosis Idiopathic Signaling Pathway
S100 Family Signaling Pathway
Cardiac Hypertrophy Signaling (Enhanced)
Pathogen Induced Cytokine Storm Signaling Pathway
Hepatic Fibrosis Signaling Pathway
Role Of Osteoclasts In Rheumatoid Arthritis Signaling Pathway
Wound Healing Signaling Pathway
Human Embryonic Stem Cell Pluripotency
IL-4 Signaling
Pulmonary Healing Signaling Pathway
-4.811
8.729
Activation Z-score

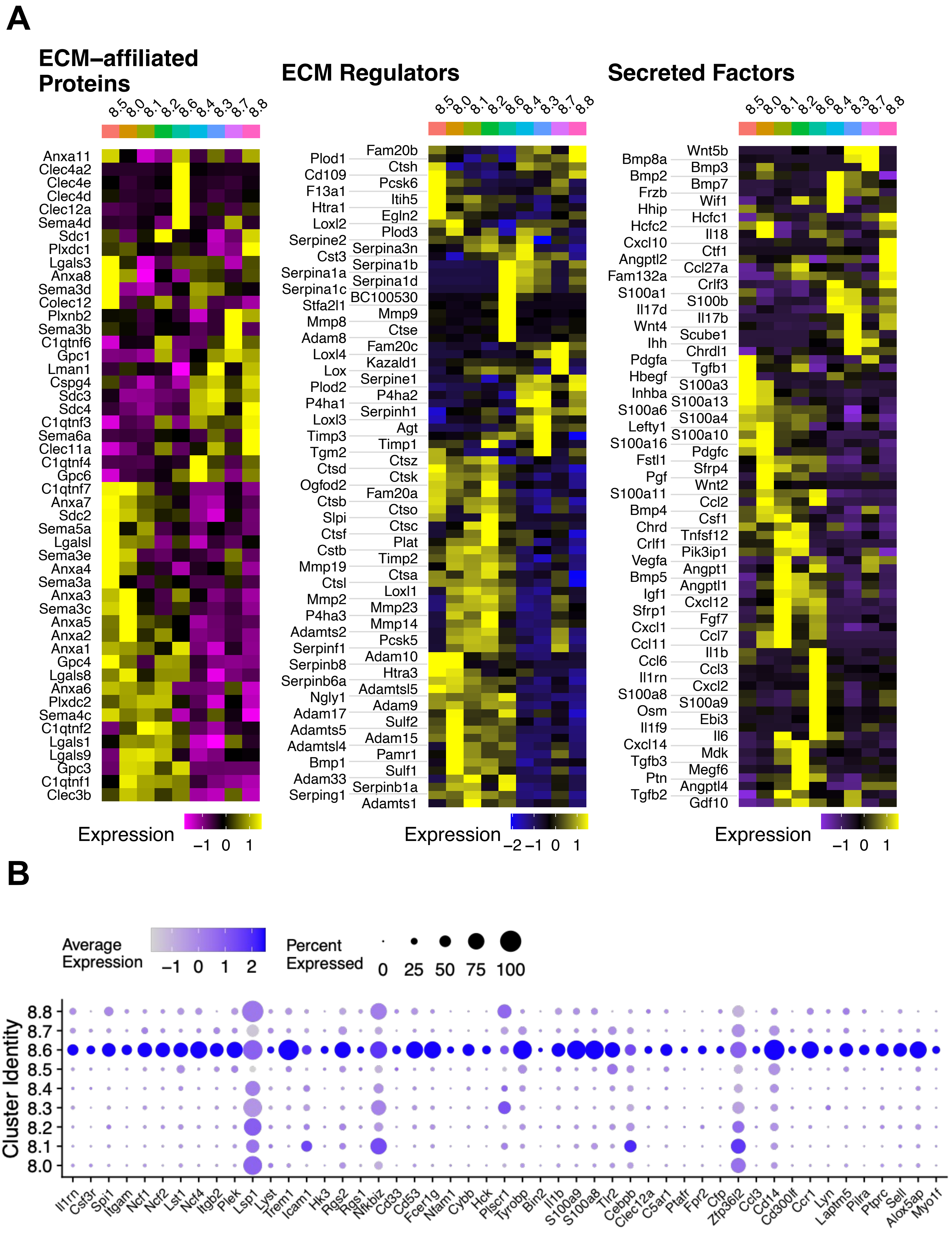
A
ECM–affiliated Proteins
8.5 8.0 8.1 8.2 8.6 8.4 8.3 8.7 8.8
Anxa11
Clec4a2
Clec4e
Clec4d
Clec12a
Sema4d
Sdc1
Plxdc1
Lgals3
Anxa8
Sema3d
Colec12
Plxnb2
Sema3b
C1qtnf6
Gpc1
Lman1
Cspg4
Sdc3
Sdc4
C1qtnf3
Sema6a
Clec11a
C1qtnf4
Gpc6
C1qtnf7
Anxa7
Sdc2
Sema5a
Lgalsl
Sema3e
Anxa4
Sema3a
Anxa3
Sema3c
Anxa5
Anxa2
Anxa1
Gpc4
Lgals8
Anxa6
Plxdc2
Sema4c
C1qtnf2
Lgals1
Lgals9
Gpc3
C1qtnf1
Clec3b
Expression
−1 0 1
ECM Regulators
8.5 8.0 8.1 8.2 8.6 8.4 8.3 8.7 8.8
Plod1
Fam20b
Cd109
Ctsh
F13a1
Pcsk6
Htra1
Itih5
Loxl2
Egln2
Serpine2
Plod3
Cst3
Serpina3n
Serpina1a
Serpina1b
Serpina1c
Serpina1d
Stfa2l1
BC100530
Mmp8
Mmp9
Adam8
Ctse
Loxl4
Fam20c
Lox
Kazald1
Plod2
Serpine1
P4ha1
P4ha2
Loxl3
Serpinh1
Timp3
Agt
Tgm2
Timp1
Ctsd
Ctsz
Ogfod2
Ctsk
Ctsb
Fam20a
Slpi
Ctso
Ctsf
Ctsc
Cstb
Plat
Mmp19
Timp2
Ctsl
Ctsa
Mmp2
Loxl1
P4ha3
Mmp23
Adamts2
Mmp14
Serpinf1
Pcsk5
Serpinb8
Adam10
Serpinb6a
Htra3
Ngly1
Adamtsl5
Adam17
Adam9
Adamts5
Sulf2
Adamtsl4
Adam15
Bmp1
Pamr1
Adam33
Sulf1
Serping1
Serpinb1a
Adamts1
Expression
−2 −1 0 1
Secreted Factors
8.5 8.0 8.1 8.2 8.6 8.4 8.3 8.7 8.8
Bmp8a
Wnt5b
Bmp2
Bmp3
Frzb
Bmp7
Hhip
Wif1
Hcfc2
Hcfc1
Cxcl10
Il18
Angptl2
Ctf1
Fam132a
Ccl27a
S100a1
Crlf3
Il17d
S100b
Wnt4
Il17b
Ihh
Scube1
Pdgfa
Chrdl1
Hbegf
Tgfb1
Inhba
S100a3
S100a6
S100a13
Lefty1
S100a4
S100a16
S100a10
Fstl1
Pdgfc
Pgf
Sfrp4
S100a11
Wnt2
Bmp4
Ccl2
Chrd
Csf1
Crlf1
Tnfsf12
Vegfa
Pik3ip1
Bmp5
Angpt1
Igf1
Angptl1
Sfrp1
Cxcl12
Cxcl1
Fgf7
Ccl11
Ccl7
Ccl6
Il1b
Il1rn
Ccl3
S100a8
Cxcl2
Osm
S100a9
Il1f9
Ebi3
Cxcl14
Il6
Tgfb3
Mdk
Ptn
Megf6
Tgfb2
Angptl4
Gdf10
Expression
−1 0 1
B
Average Expression
−1 0 1 2
Percent Expressed
0 25 50 75 100
Cluster Identity
8.8
8.7
8.6
8.5
8.4
8.3
8.2
8.1
8.0
Il1rn
Csf3r
Spi1
Itgam
Ncf1
Ncf2
Lst1
Ncf4
Itgb2
Plek
Lsp1
Lyst
Trem1
Icam1
Hk3
Rgs2
Rgs1
Nfkbiz
Cd33
Cd53
Fcer1g
Nfam1
Cybb
Hck
Plscr1
Tyrobp
Bin2
Il1b
S100a9
S100a8
Tlr2
Cebpb
Clec12a
C5ar1
Ptafr
Fpr2
Cfp
Zfp36l2
Ccl3
Cd14
Cd300lf
Ccr1
Lyn
Laptm5
Pilra
Ptprc
Sell
Alox5ap
Myo1f

SFig 4

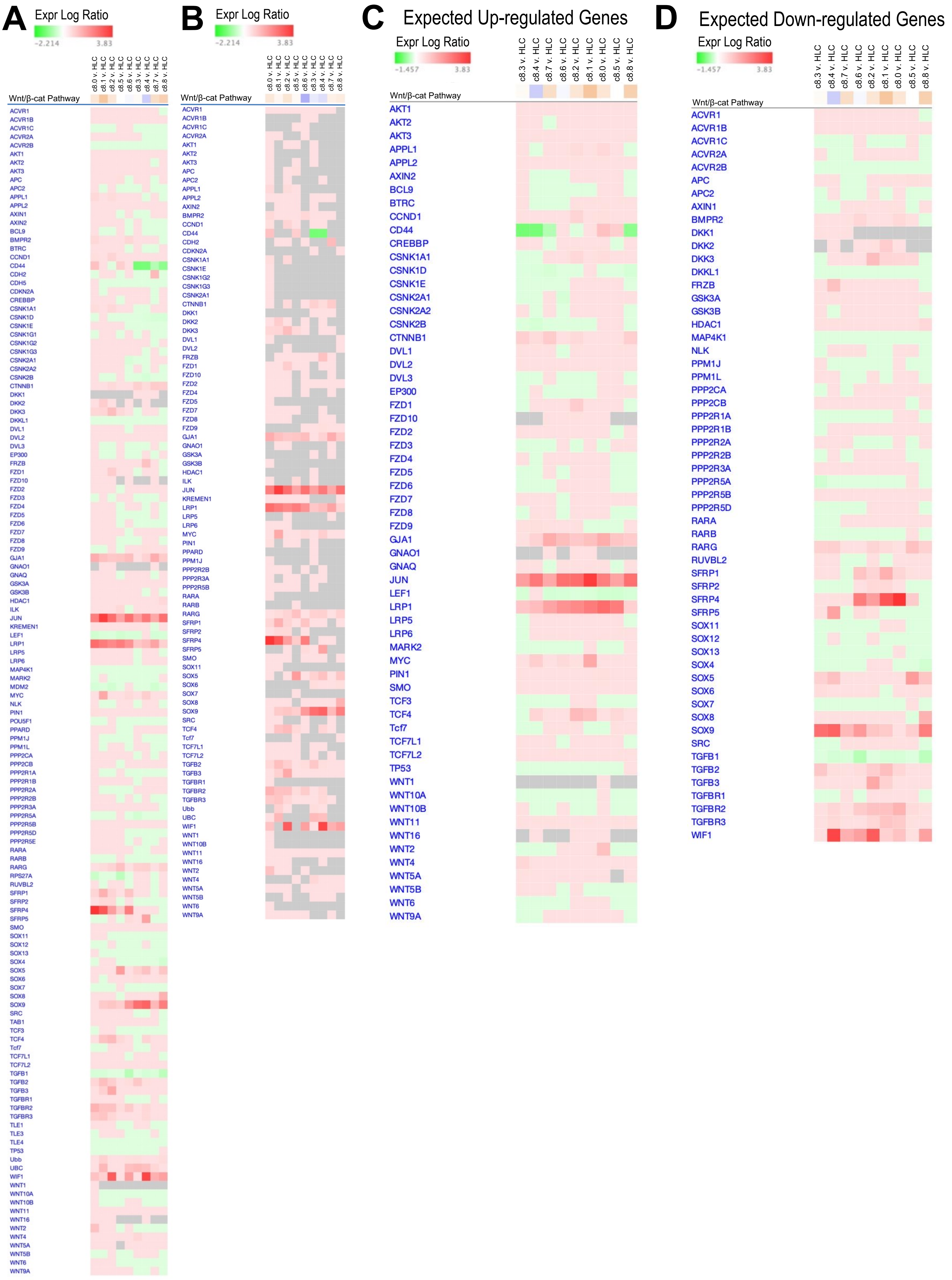
A
Expr Log Ratio
-2.214
3.83
Wnt/β-cat Pathway
B
Expr Log Ratio
-2.214
3.83
Wnt/β-cat Pathway
C
Expected Up-regulated Genes
Expr Log Ratio
-1.457
3.83
c8.3 v. HLC
c8.4 v. HLC
c8.7 v. HLC
c8.6 v. HLC
c8.2 v. HLC
c8.1 v. HLC
c8.0 v. HLC
c8.5 v. HLC
c8.8 v. HLC
Wnt/β-cat Pathway
AKT1
AKT2
AKT3
APPL1
APPL2
AXIN2
BCL9
BTRC
CCND1
CD44
CREBBP
CSNK1A1
CSNK1D
CSNK1E
CSNK2A1
CSNK2A2
CSNK2B
CTNNB1
DVL1
DVL2
DVL3
EP300
FZD1
FZD10
FZD2
FZD3
FZD4
FZD5
FZD6
FZD7
FZD8
FZD9
GJA1
GNAO1
GNAQ
JUN
LEF1
LRP1
LRP5
LRP6
MARK2
MYC
PIN1
SMO
TCF3
TCF4
Tcf7
TCF7L1
TCF7L2
TP53
WNT1
WNT10A
WNT10B
WNT11
WNT16
WNT2
WNT4
WNT5A
WNT5B
WNT6
WNT9A
D
Expected Down-regulated Genes
Expr Log Ratio
-1.457
3.83
c8.3 v. HLC
c8.4 v. HLC
c8.7 v. HLC
c8.6 v. HLC
c8.2 v. HLC
c8.1 v. HLC
c8.0 v. HLC
c8.5 v. HLC
c8.8 v. HLC
Wnt/β-cat Pathway
ACVR1
ACVR1B
ACVR1C
ACVR2A
ACVR2B
APC
APC2
AXIN1
BMPR2
DKK1
DKK2
DKK3
DKKL1
FRZB
GSK3A
GSK3B
HDAC1
MAP4K1
NLK
PPM1J
PPM1L
PPP2CA
PPP2CB
PPP2R1A
PPP2R1B
PPP2R2A
PPP2R2B
PPP2R3A
PPP2R5A
PPP2R5B
PPP2R5D
RARA
RARB
RARG
RUVBL2
SFRP1
SFRP2
SFRP4
SFRP5
SOX11
SOX12
SOX13
SOX4
SOX5
SOX6
SOX7
SOX8
SOX9
SRC
TGFB1
TGFB2
TGFB3
TGFBR1
TGFBR2
TGFBR3
WIF1

SFig 5

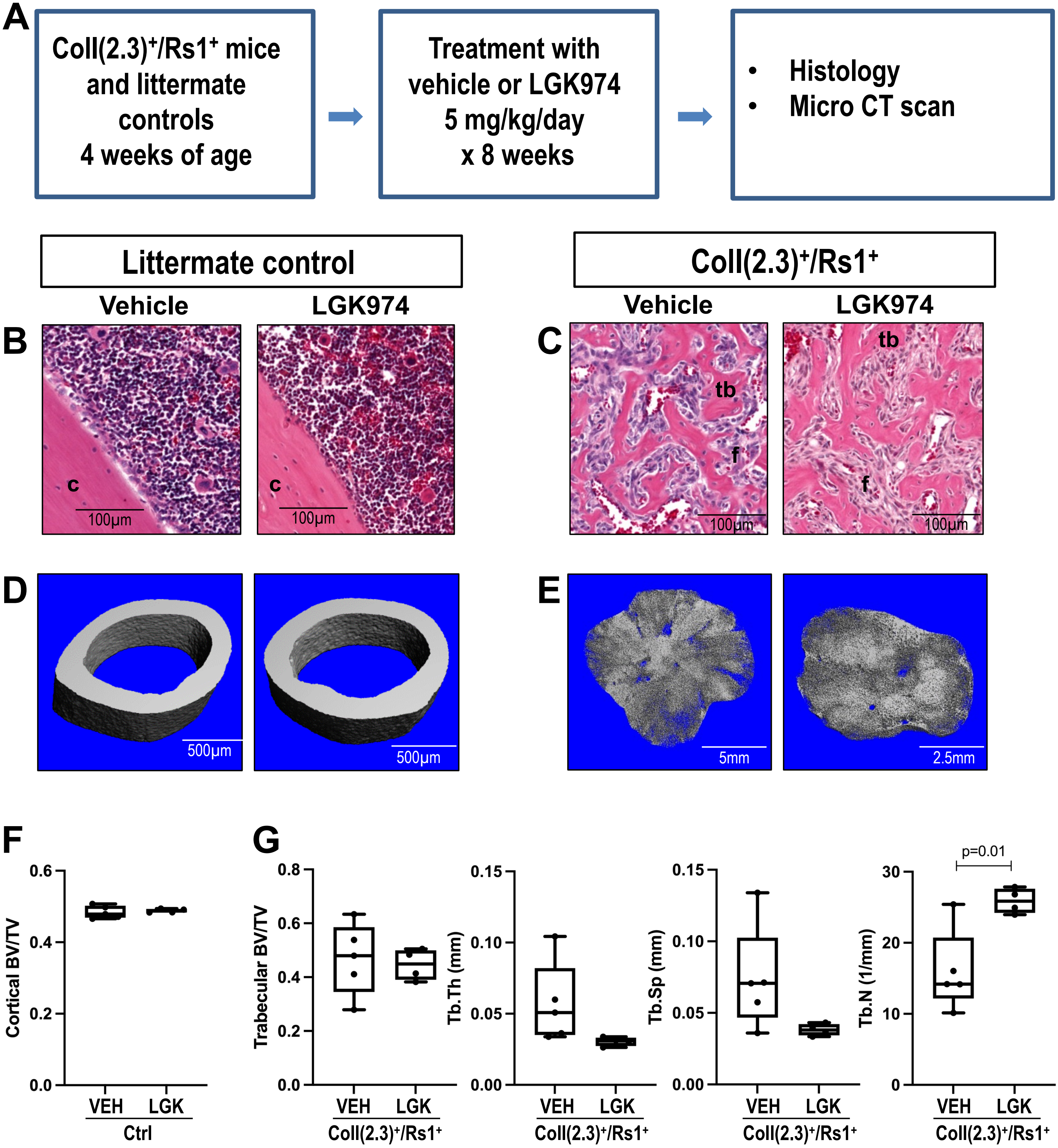
A
Coll(2.3)+/Rs1+ mice and littermate controls 4 weeks of age
Treatment with vehicle or LGK974 5 mg/kg/day x 8 weeks
Histology
Micro CT scan
Littermate control
Coll(2.3)+/Rs1+
Vehicle
LGK974
B
C
c
tb
f
100µm
D
E
500µm
5mm
2.5mm
F
G
Cortical BV/TV
Trabecular BV/TV
Tb.Th (mm)
Tb.Sp (mm)
Tb.N (1/mm)
p=0.01
VEH
LGK
Ctrl
Coll(2.3)+/Rs1+

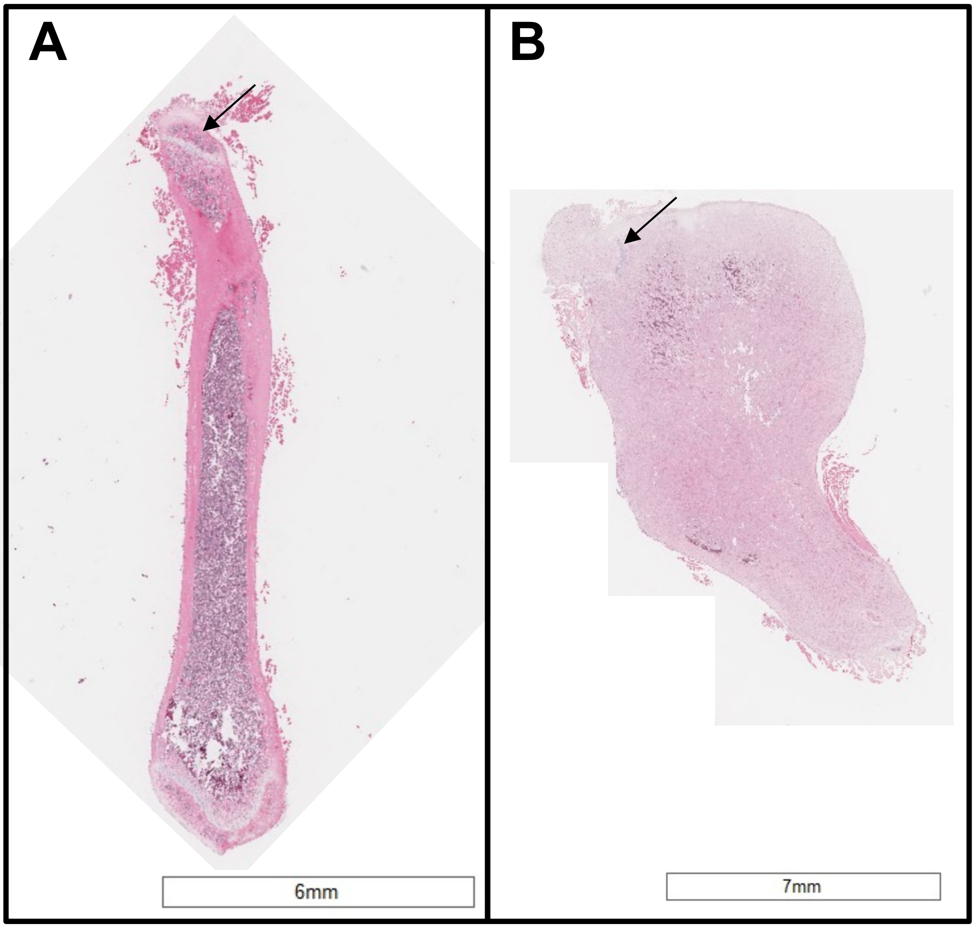
A
6mm
B
7mm

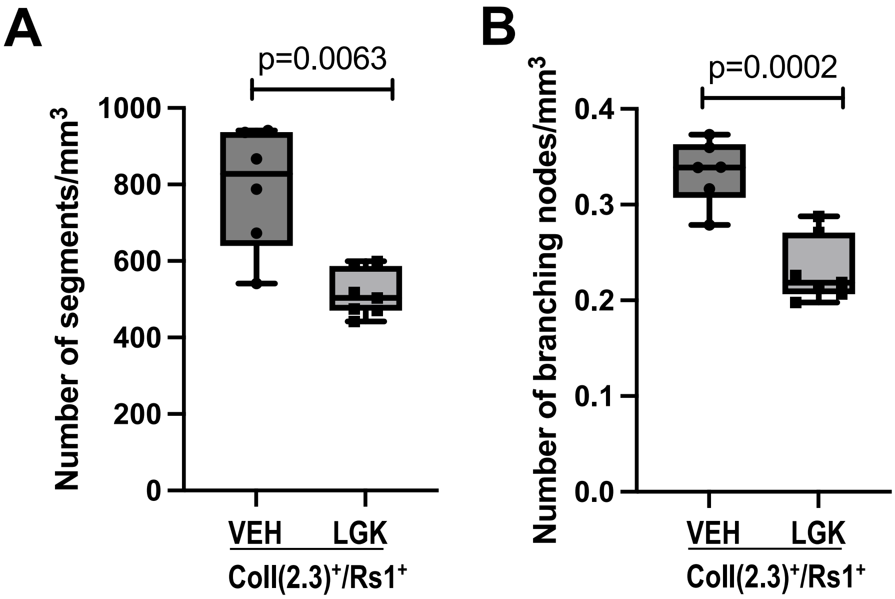
A
p=0.0063
Number of segments/mm3
1000
800
600
400
200
0
VEH
LGK
Coll(2.3)+/Rs1+
B
p=0.0002
Number of branching nodes/mm3
0.4
0.3
0.2
0.1
0.0
VEH
LGK
Coll(2.3)+/Rs1+

A. Osterix (Sp7)

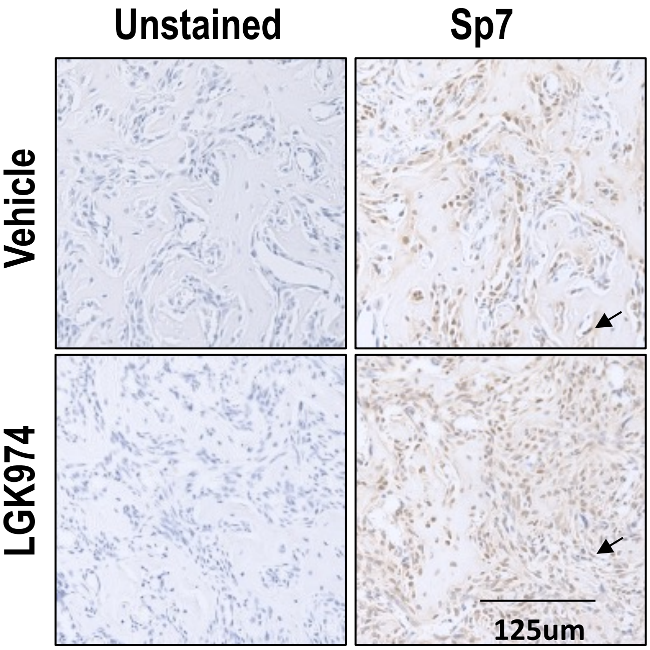

B. Osteocalcin (OCN)

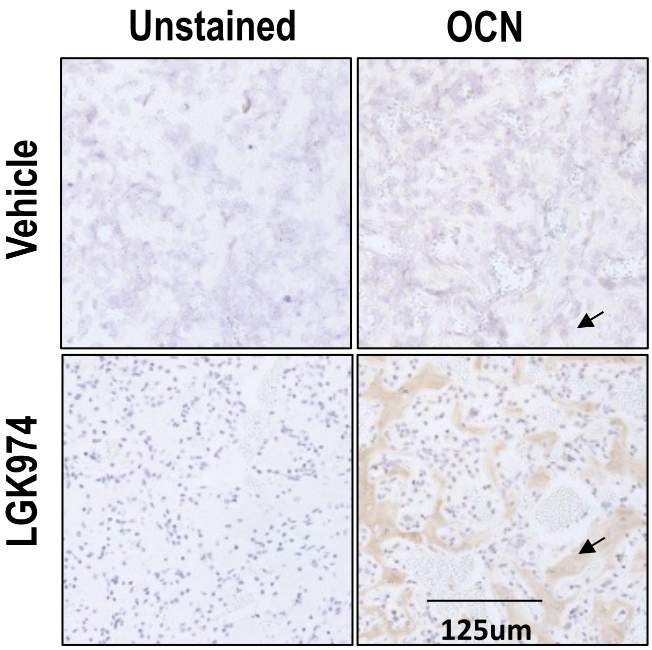

C. Cathepsin K (CTSK)

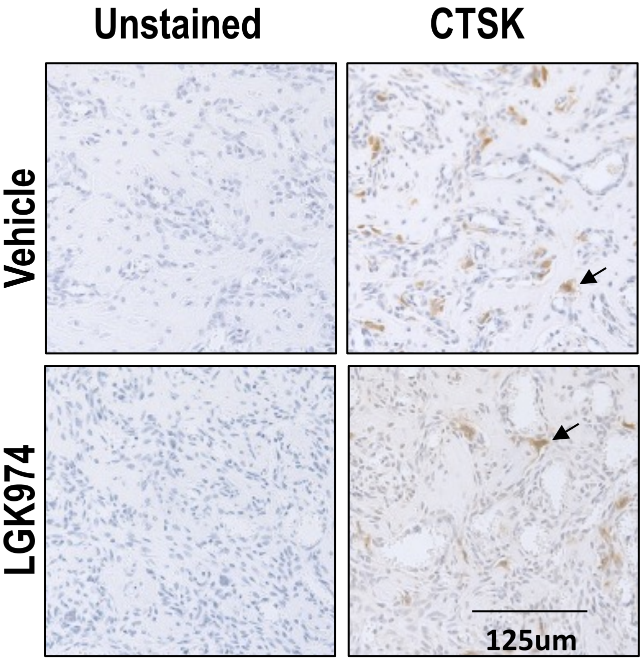

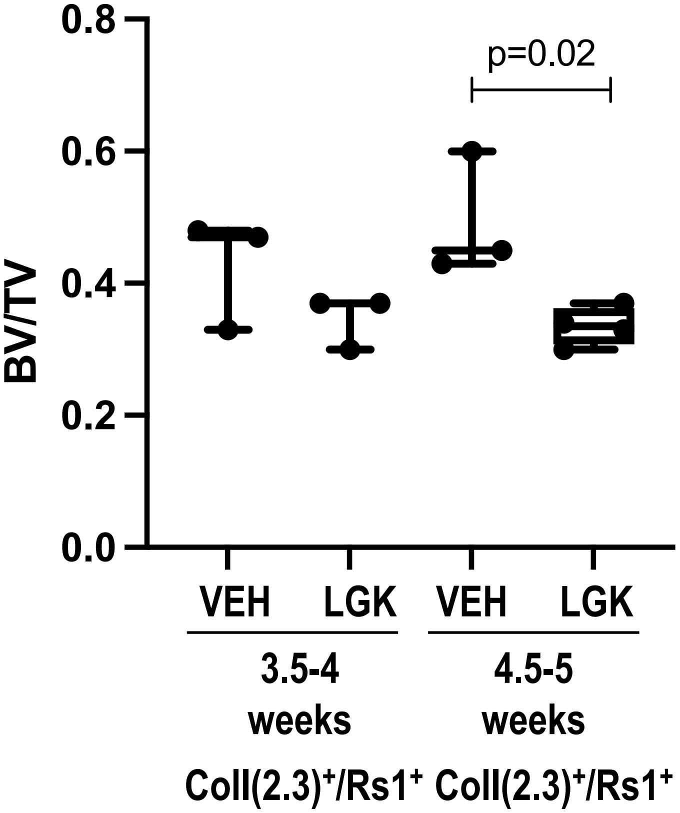

0.8
0.6
0.4
0.2
0.0
BV/TV
p=0.02
VEH
LGK
VEH
LGK
3.5-4 weeks
4.5-5 weeks
Coll(2.3)+/Rs1+
Coll(2.3)+/Rs1+

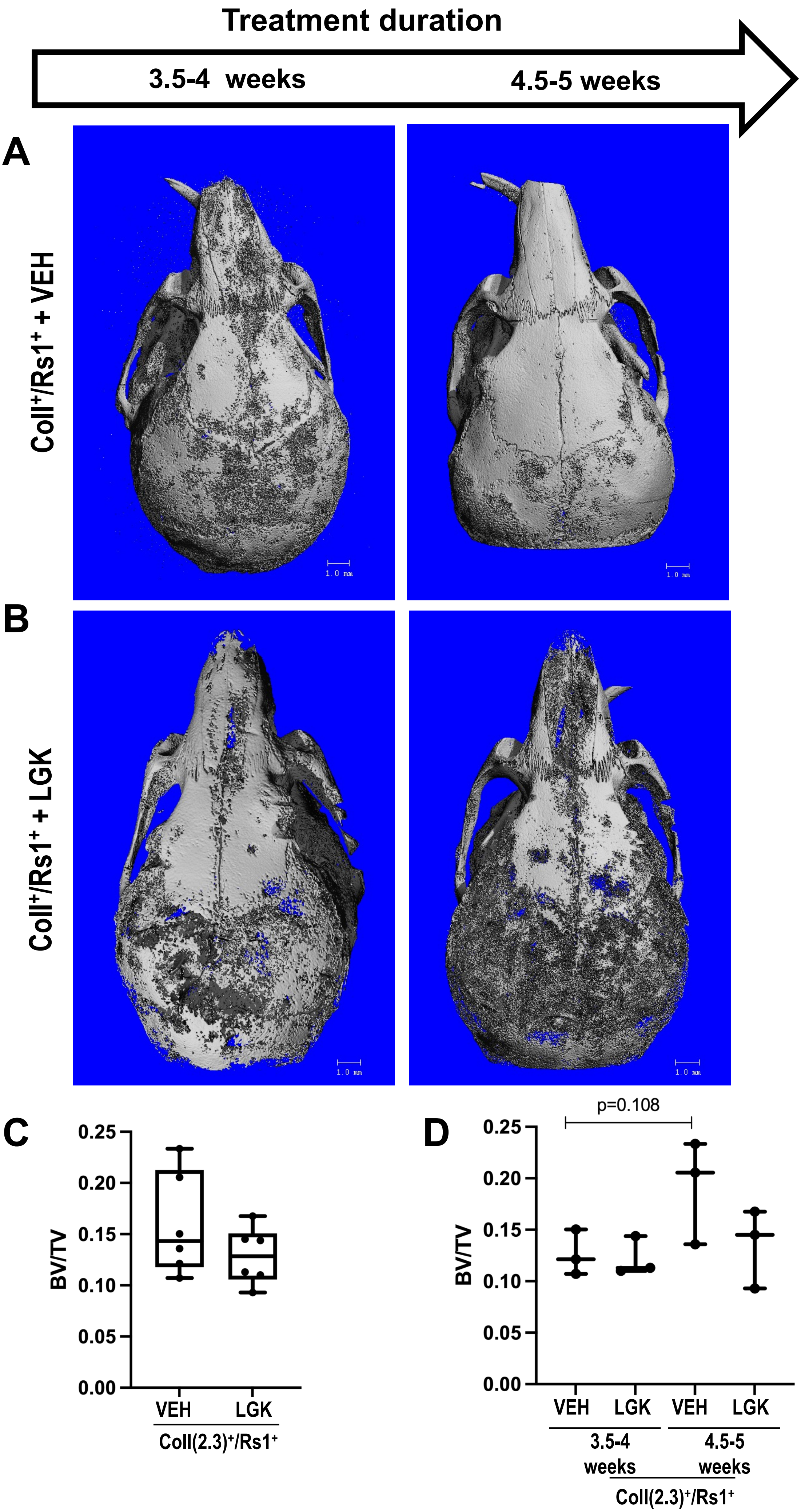
Treatment duration
3.5-4 weeks
4.5-5 weeks
A
Coll⁺/Rs1⁺ + VEH
B
Coll⁺/Rs1⁺ + LGK
1.0 mm
C
BV/TV
0.25
0.20
0.15
0.10
0.05
0.00
VEH
LGK
Coll(2.3)⁺/Rs1⁺
D
p=0.108
BV/TV
0.25
0.20
0.15
0.10
0.05
0.00
VEH
LGK
VEH
LGK
3.5-4 weeks
4.5-5 weeks
Coll(2.3)⁺/Rs1⁺

SFig 11

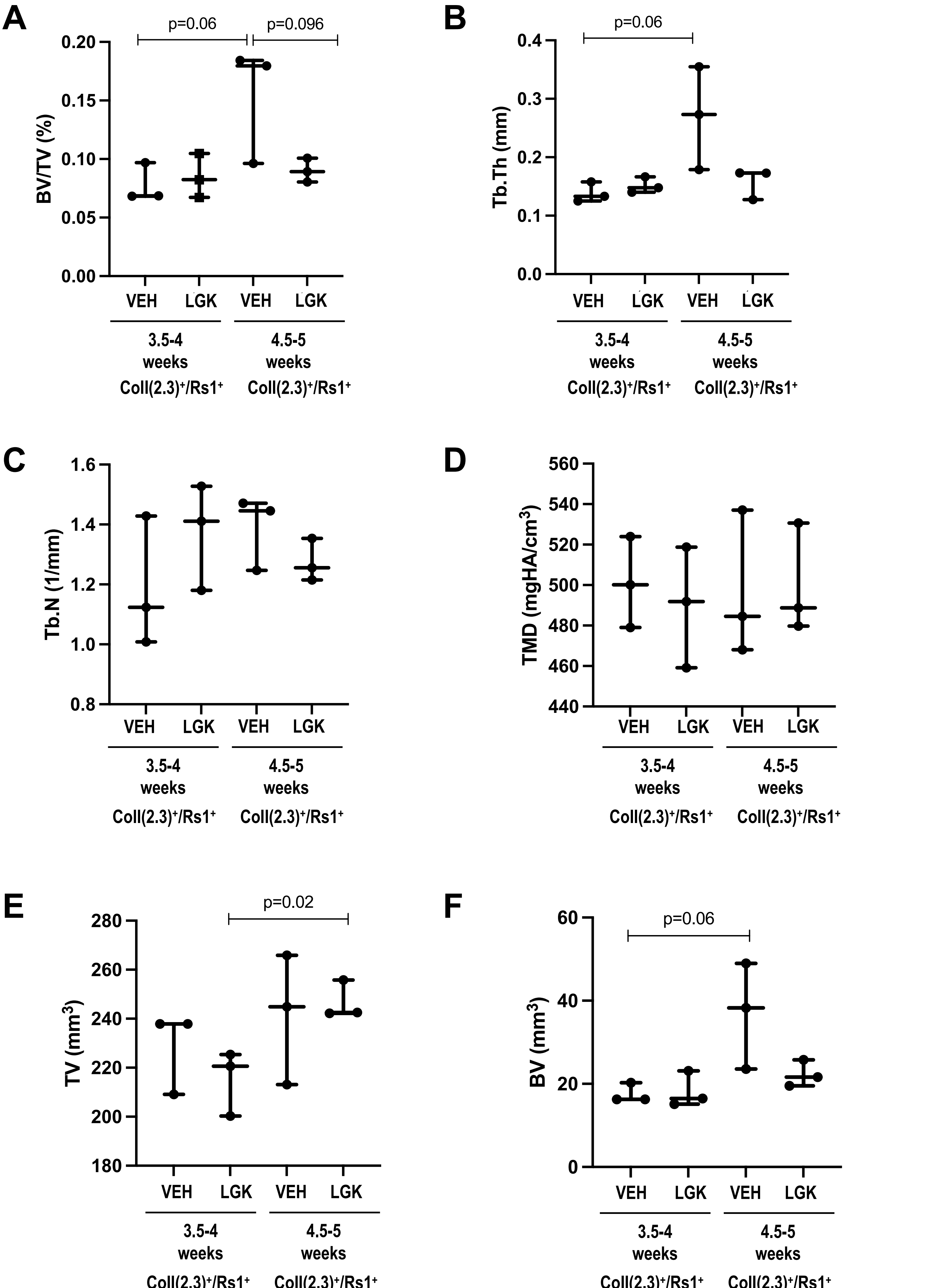
A
p=0.06
p=0.096
BV/TV (%)
0.20
0.15
0.10
0.05
0.00
VEH
LGK
VEH
LGK
3.5-4 weeks Coll(2.3)+/Rs1+
4.5-5 weeks Coll(2.3)+/Rs1+
B
p=0.06
Tb.Th (mm)
0.4
0.3
0.2
0.1
0.0
C
Tb.N (1/mm)
1.6
1.4
1.2
1.0
0.8
D
TMD (mgHA/cm3)
560
540
520
500
480
460
440
E
p=0.02
TV (mm3)
280
260
240
220
200
180
F
p=0.06
BV (mm3)
60
40
20
0
SFig 12

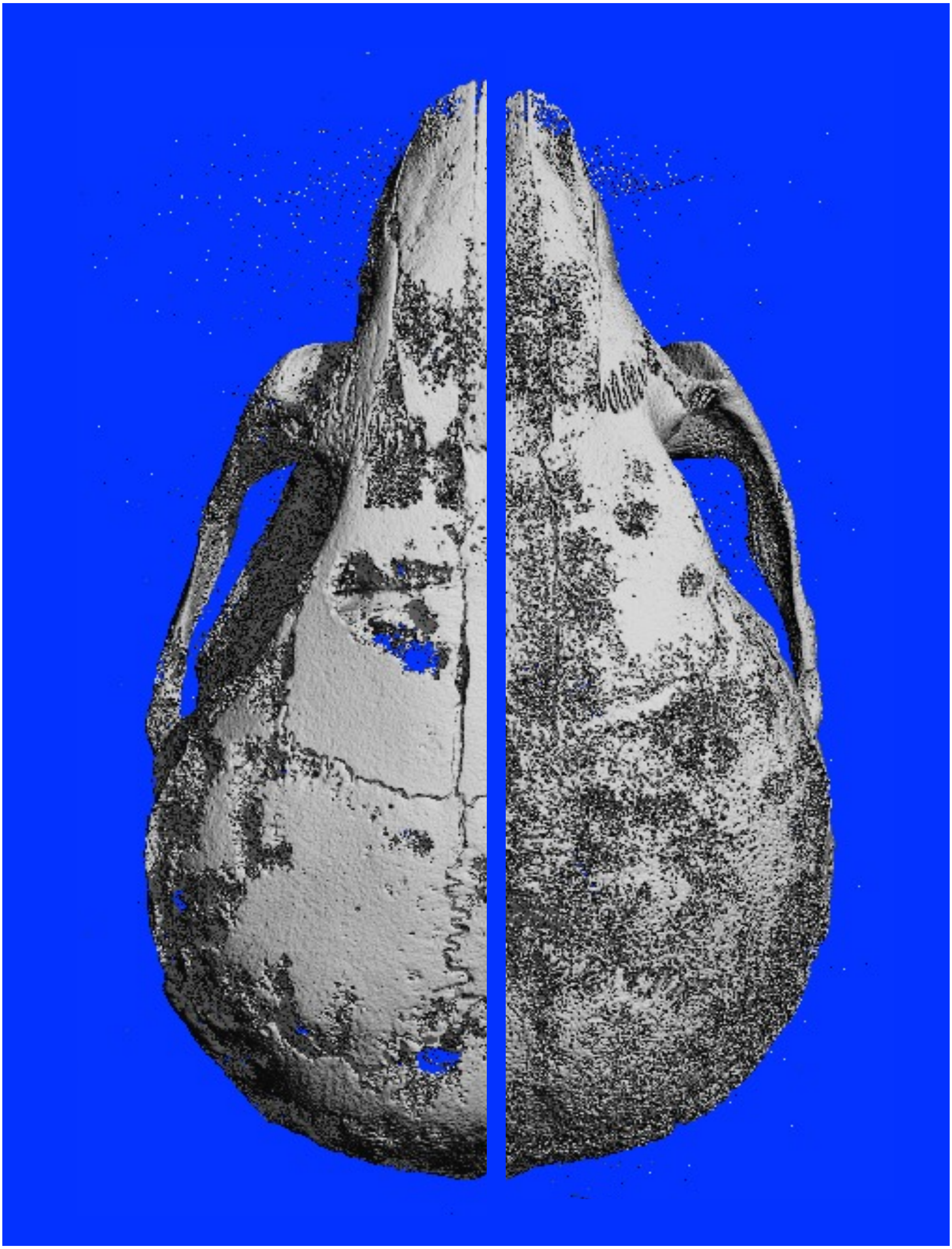

Cover Figure Submission
